# Altered Gene Expression Within the Renin–Angiotensin System in Normal Aging and Dementia

**DOI:** 10.1093/gerona/glad241

**Published:** 2023-10-09

**Authors:** Hannah M Tayler, Robert MacLachlan, Özge Güzel, Robert A Fisher, Olivia A Skrobot, Mohamed A Abulfadl, Patrick G Kehoe, J Scott Miners

**Affiliations:** Dementia Research Group, Clinical Neurosciences, Bristol Medical School, University of Bristol, Bristol, UK; Dementia Research Group, Clinical Neurosciences, Bristol Medical School, University of Bristol, Bristol, UK; Dementia Research Group, Clinical Neurosciences, Bristol Medical School, University of Bristol, Bristol, UK; Dementia Research Group, Clinical Neurosciences, Bristol Medical School, University of Bristol, Bristol, UK; Dementia Research Group, Clinical Neurosciences, Bristol Medical School, University of Bristol, Bristol, UK; Dementia Research Group, Clinical Neurosciences, Bristol Medical School, University of Bristol, Bristol, UK; Dementia Research Group, Clinical Neurosciences, Bristol Medical School, University of Bristol, Bristol, UK; Dementia Research Group, Clinical Neurosciences, Bristol Medical School, University of Bristol, Bristol, UK; (Biological Sciences Section)

**Keywords:** Angiotensin-converting enzyme (ACE), Angiotensin-II type 1 receptor (*AGT1R*), Angiotensin-II type 2 receptor (*AGT2R*)

## Abstract

The renin–angiotensin system (RAS) is dysregulated in Alzheimer’s disease (AD). In this study, we have explored the hypothesis that an ­age-­related imbalance in brain RAS is a trigger for RAS dysregulation in AD. We characterized RAS gene expression in the frontal cortex from (i) a cohort of normal aging (*n* = 99, age range = 19–96 years) and (ii) a case–control cohort (*n* = 209) including AD (*n* = 66), mixed dementia (VaD + AD; *n* = 50), pure vascular dementia (VaD; *n* = 42), and age-matched controls (*n* = 51). The AD, mixed dementia, and age-matched controls were further stratified by Braak tangle stage (BS): BS0–II (*n* = 48), BSIII–IV (*n* = 44), and BSV–VI (*n* = 85). Gene expression was calculated by quantitative PCR (qPCR) for *ACE1*, *AGTR1*, *AGTR2*, *ACE2*, *LNPEP*, and *MAS1* using the 2^−∆∆Cq^ method, after adjustment for reference genes (*RPL13* and *UBE2D2*) and cell-specific calibrator genes (*NEUN*, *GFAP*, *PECAM*). *ACE1* and *AGTR1*, markers of classical RAS signaling, and *AGTR2* gene expression were elevated in normal aging and gene expression in markers of protective downstream regulatory RAS signaling, including *ACE2*, *MAS1*, and *LNPEP*, were unchanged. In AD and mixed dementia, *AGTR1* and *AGTR2* gene expression were elevated in BSIII–IV and BSV–VI, respectively. *MAS1* gene expression was reduced at BSV–VI and was inversely related to parenchymal Aβ and tau load. *LNPEP* gene expression was specifically elevated in VaD. These data provide novel insights into RAS signaling in normal aging and dementia.

Chronically overactivated renin–angiotensin system (RAS) signaling is implicated in age-related diseases, including cardiac and renal disease, and neurodegenerative diseases, including Alzheimer’s disease (AD; reviewed (1)). Classical RAS (cRAS) signaling contributes to a pro-oxidative and inflammatory state associated with aging (reviewed (2)). Disruption of the *Agtr1a* gene diminishes cRAS signaling and attenuates oxidative stress promoting longevity in mice (3) and polymorphisms in the *AGTR1* gene are linked to longevity in humans (4). Life-long angiotensin-II type 1 receptor (AT1R) blockers protect against age-related dysfunction and damage to the heart, kidney, and brain (5).

Overactive cRAS signaling is linked to central nervous system (CNS) disorders, including anxiety and depression (6), cognitive impairment (7), and neurodegenerative diseases, including Parkinson’s disease and AD (8). RAS signaling has localized pleiotropic effects within the brain, independent of blood pressure (BP) regulation, which potentially influence pathological processes that underpin neurodegenerative diseases, including neuroinflammation, oxidative stress, mitochondrial and cholinergic dysfunction (reviewed (8)). Our previous studies, and findings from others, indicate that cRAS signaling is overactivated in AD and angiotensin-converting enzyme-1 (ACE-1), angiotensin-II (ang-II), and ang-II type 1 receptor (AT1R) levels are elevated (9–12). Incontrast, protective regulatory RAS (rRAS) pathways, including ACE-2/Ang-(1–7)/MasR and ang-III/ang-IV/AT4R signaling, are defective in AD (13). Increased ACE-1:ACE-2 ratio is related to higher parenchymal Aβ and tau load (13) and overactive AT1R signaling correlates with oxidative stress, elevated Aβ and tau, and impaired cognition in AD (14).

Whether age-related changes in RAS predispose individuals to an imbalance in RAS signaling in AD is unclear. We recently reported age- and disease-related changes in the expression and activity of ACE-1, and ang-II protein levels, suggesting that RAS signaling in the early stages of AD differed from disrupted RAS signaling as a result of normal aging (15). Due to concerns over the specificity of commercially available antibodies (16–18), the expression of angiotensin peptide receptors in both aging and AD remains undetermined. In this study, we have therefore measured the gene expression of *ACE1* and *ACE2*, and the RAS receptors *AGTR1*, *AGTR2*, and *MAS1*, in normal aging and an independent case–control study comprising pure AD, mixed AD/VaD (mixed dementia), and vascular dementia (VaD), stratified according to Braak tangle stage as a marker of disease stage in AD.

## Method

### Brain Tissue

Postmortem human brain tissue was obtained from the South West Dementia Brain Bank (SWDBB; University of Bristol, UK), the Edinburgh Brain and Tissue Bank (EBTB; University of Edinburgh, UK), and the Newcastle Brain Tissue Resource (NBTR; Newcastle University, UK). The study was approved by the SWDBB management committee (Human Tissue Authority license number 12273) under the terms approved by the Bristol (18/SW/0029) and Edinburgh (16/ES/0084) ethical research committees. For brain donations, the right cerebral hemisphere had previously been fixed in formalin for 3 weeks. The left cerebral hemisphere had been sliced and stored frozen at −80°C. We studied brain tissue from the frontal cortex (Brodmann area 9).

### Cohorts

The normal aging cohort (*n* = 99) included donors from 19 to 96 years of age without a clinical history of cognitive impairment and absence of neuropathological abnormalities. This cohort was stratified into quartiles to generate a similar number of cases in each age group: <52 years (*n* = 25), 55–71 (*n* = 25), 72–85 (*n* = 25), and 86 years and older (*n* = 24). The demographic summary of the aging cohort is shown in Table 1A.

**Table 1.** Demographic summaries of the aging, case-control and Braak tangle stage cohorts

A. Normal aging cohort (*n* = 99)				
Quartile: ages	Q1: <52 y	Q2: 52–71 y	Q3: 72–85 y	Q4: 86+ y
(*n*)	*n* = 25	*n* = 25	*n* = 25	*n* = 24
Age (y)	44 (40–49)	65 (61–69)	78 (74–82)	91 (87–93)
Sex (F:M)	8:17	8:17	10:14	13:11
Postmortem delay (h)	86 (58–95)	69 (47–96)	47 (36–61)	42 (31–57)
Brain bank: SWDBB/EBTB	1/24	5/20	21/4	42/-
Hypertensive: Y/N/-	2/20/3	13/12/0	19/5/1	21/3/0
RAS blocker total (ACEi/ARB)	1 (1/0)	5 (5/0)	11 (9/7)	11 (10/4)

B. Case–control cohort (*n* = 209)				
Case–control group	Controls	AD	Mixed	VaD
(*n*)	*n* = 51	*n* = 66	*n* = 50	*n* = 42
Age (y)	85 (77–90)	81 (75–87)	86 (81–90)	84 (77–90)
Sex (F:M)	25:26	35:31	31:19	17:25
Postmortem delay (h)	41 (32–58)	35 (21–51)	36 (26–47)	42 (27–59)
Age of dementia onset (y)	-	73 (65–79)	78 (73–84)	80 (74–84)
Duration of dementia (y)	-	8 (5–11)	7 (5–11)	4 (2–7)
Braak stage: 0–II/III–IV/V–VI	42/9/-	-/16/50	1/14/35	29/12/-
Brain bank: SWDBB/NBTR	51/0	66/0	50/0	21/21
Hypertensive: Y/N/-	45/5/1	55/9/2	38/7/5	34/6/2
RAS blocker total (ACEi/ARB)	24 (22/10)	16 (14/3)	16 (16/6)	10 (10/2)

C. Braak tangle stage cohort (*n* = 177; all SWDBB)				
Braak tangle stage group	BS0**–**II	BSIII**–**IV	BSV**–**VI	
(*n*)	*n* = 48	*n* = 44	*n* = 85	
Age (y)	86 (77–92)	86 (82–91)	81 (75–87)	
Sex (F:M)	22:26	28:16	46:39	
Postmortem delay (h)	42 (30–58)	38 (26–67)	35 (22–50)	
Hypertensive: Yes/No/-	44/4/0	34/8/2	68/11/6	
RAS blocker total (ACEi/ARB)	24 (22/9)	14 (14/2)	22 (20/8)	

*Notes*: ACEi = ACE inhibitor; AD = Alzheimer’s disease; ARB = angiotensin-receptor blocker; BS = Braak tangle stage; EBTB = Edinburgh Brain and Tissue Bank; Mixed = mixed Alzheimer’s/vascular dementia; NBTR = Newcastle Brain Tissue Resource; RAS = renin–angiotensin system; SWDBB = South West Dementia Brain Bank; VaD = vascular dementia. Age and postmortem delay are presented as median and interquartile range. Age of dementia onset and duration of dementia were not available for *n* = 2 AD, *n* = 1 mixed, and *n* = 2 VaD cases. Hypertensive status was available for the majority, but not all cases (-). The total number of hypertensive cases known to have received RAS-targeting medications is shown. A breakdown of case numbers on ACE inhibitors and angiotensin-II receptor blockers are also given for each group; noting the discrepancy between the total number and breakdown by medication subtype, some individuals had received treatment with both medication types at different times.

A case–control cohort (*n* = 209) included age-matched controls (*n* = 51) without a history of dementia, few or absent neuritic plaques, a Braak tangle stage of III or less, and no other neuropathological abnormalities. AD cases (*n* = 66) had a clinical diagnosis of dementia and an intermediate or high level of AD neuropathological change according to the National Institute on Aging and Alzheimer's Association (NIA–AA) guidelines (19). VaD cases (*n* = 42) had a clinical history of dementia and neuropathological changes including multiple infarcts/ischemic lesions and moderate to severe atheroma and/or arteriosclerosis and only occasional neuritic plaques and mixed AD/VaD cases (*n* = 50) had neuropathological changes consistent with AD, plus multiple infarcts/ischemic lesions and moderate to severe atheroma and/or arteriosclerosis. The demographics of the case–control dementia cohort are presented in Table 1B.

A cohort comprising the AD, mixed, and age-matched controls (n = 167), was combined with a small number of additonal cases from the SWDBB (n = 10), and stratified according to Braak tangle stage into the following groups: Braak stage 0–II cases (*n* = 48), Braak stage III–IV cases (*n* = 44), and Braak stage V–VI cases (*n* = 85). The demographics of the Braak tangle stage cohort are shown in Table 1C.

In this study, hypertension was defined as (i) a diagnosis of essential hypertension or (ii) a retrospective classification of systolic BP ≥ 140 mm Hg and/or diastolic BP ≥ 90 mm Hg noted on at least 2 separate occasions within the donor’s medical records. Any recorded use of either ACE inhibitors or ang-II receptor blockers in clinical records (any length of time/any age) was used to identify donors with a history of RAS medication.

Parenchymal Aβ and phospho-tau loads were previously determined (20). In brief, formalin-fixed paraffin-­embedded (FFPE) sections of frontal cortex underwent immunohistochemical labeling for pan-Aβ 4G8 antibody (1:8000) or an AT8 phospho-tau antibody (1:500) using a Ventana BenchMark ULTRA automated immunostainer (Roche Tissue Diagnostics, Oro Valley, AZ, U.S.A). Field-fraction analysis was used to determine the area of the section positive for the target antigen using image analysis software (Image-Pro Plus 7, Media Cybernetics, Rockville, MD, U.S.A).

A list of UK brain bank network identifier numbers for cases used in this study is shown in Supplementary Table 1.

### RNA Extraction and Reverse Transcription PCR (RT-PCR)

RNA was extracted from fresh frozen samples of frontal cortex (70 mg) using the RNeasy Lipid Tissue Mini Kit (Qiagen, Hilden, Germany), including DNase I treatment (RNase-free DNase set, Qiagen). RNA was quantified using Qubit RNA BR Assay Kit (Thermo Fisher Scientific, Waltham, MN, U.S.A) and RNA purity determined by spectroscopic measurement at 260 nm (and the absorbance of potential contaminants at 280 or 230 nm; NanoPhotometer, Implen, Munich, Germany).

RNA integrity (RIN) was measured with an Agilent 2100 Bioanalyzer using the Agilent 6000 RNA Nano Kit. RIN values ranged from 2.1 to 7.1 across the entire cohort (*n* = 216). RIN values were unchanged relative to age across the normal aging cohort (Supplementary Figure 1A) but were weakly positively correlated to tissue pH (a marker of tissue integrity) accounting for ~13% of the variation (*r*^2^ = 0.13; *p* < .0001). RIN values were also significantly lower in the AD/mixed group compared to age-matched controls (and VaD cases; Supplementary Figure 1B) and in BSV–VI compared to BS0–II (Supplementary Figure 1C).

RNA, diluted to 0.2 µg/µL, was converted to cDNA by High-Capacity cDNA Reverse Transcription Kit (Applied Biosystems, Waltham, MN, U.S.A), according to the standard protocol, with the inclusion of an RNase inhibitor. qPCR reactions (10 µL) for each of the reference genes (RGs), cell-type markers, and target genes were prepared in triplicate including cDNA, TaqMan Fast Advanced Master Mix, and TaqMan assay primers (Applied Biosystems). Quantitative PCR (qPCR) reactions were performed in the ViiA 7 Real-Time PCR System (Thermo Fisher Scientific) with polymerase activation (hold 95°C for 2 seconds), PCR × 40 cycles of denature (95°C for 1 second), and anneal/extend (60°C for 20 seconds).

Gene expression was calculated in each sample from the difference in threshold cycle (Cq) between the target and RG (combined mean of *UBE2D2* and *RPL13*) or cell-type calibrators: *NEUN*, *GFAP*, or *PECAM1*. The 2 ubiquitously expressed references genes, *UBE2D2* and *RPL13*, were chosen based on findings of Rydbirk et al. (21) who demonstrated that they were among the most stable RGs in human tissue across various neurodegenerative diseases including AD. The primers and probes used in the study are listed in Supplementary Tables 2 and 3, respectively. The relative quantitative 2^−∆∆Cq^ method (22) was used to calculate the fold change in target gene expression relative to the mean expression of the control group defined as (i) the youngest age quartile (<52 years) for the normal aging cohort, (ii) the age-matched controls in the case–control cohort, and (iii) Braak stage 0–II for the Braak cohort.

### Triple Immunofluorescence Labeling of ACE-1 and ACE-2

FFPE–brain tissue sections (Brodmann area 9; frontal lobe) from a 92-year-old non-AD control donor (MRC identifier BBN006.34115) with a postmortem delay of 76.5 hours was obtained from the original wax-embedded block used for diagnosis to minimize time spent in formalin (less than 3 weeks prior to fixation). Sections were incubated at 65°C for 2 hours, dewaxed and cleared in ethanol, and washed in running water to remove excess wax. Microwave epitope retrieval pretreatment in Tris ethylenediaminetetraacetic acid (EDTA) pH 9.0 for 10 minutes, followed by a phosphate buffered saline (PBS) wash and a further wash in running water was performed. Brain tissue was permeabilized with 0.1% Triton X-100 and blocked in 5% normal donkey serum (NDS) followed by overnight incubation at 4°C with primary antibodies diluted in 0.5% NDS: ACE-1 at 1 in 50 (AF929, R&D Systems, Minneapolis, MN, U.S.A), ACE-2 at 1 in 200 (Ab15348, Abcam), and glial acidic fibrillary protein - GFAP at 1 in 500 (Ab4674, Abcam). After ×4 washes in PBS, sections were incubated for 1 hour at room temperature (RT) with Alexa Fluor secondary antibodies donkey anti-goat 546 (A11056, Invitrogen, Waltham, MA, U.S.A) and donkey anti-rabbit 488 (Ab150073, Abcam, Cambridge, U.K). Sections were washed in PBS and incubated with Alexa Fluor goat anti-chicken 405 (A48260, Invitrogen) for 1 hour. After another PBS wash, SudanBlack (Millipore, Burlington, MN, U.S.A) was applied for 5 minutes, and washed until clear in 70% ethanol. Slides were mounted using mounting medium (Vectashield, Vector Labs, Newark, CA, U.S.A). Images were captured using a Nikon C1 confocal microscope (×60 objective, oil) at a resolution of 1024 × 1024 px.

### In Situ Hybridization (RNAscope) Analysis of Cell-Specific Distribution of AGTR1, AGTR2, and MAS1

In situ hybridization was performed using the RNAscope Multiplex Fluorescent Reagent Kit v2 Assay (323100, Advanced Cell Diagnostics, Newark, CA, U.S.A) following manufacturer’s guidelines. Briefly, FFPE sections from the same non-AD age-matched control brain used for IF-labeling (MRC identifier BBN006.34115) were dried at 60°C for 1 hour before being deparaffinized in clearene and 100% ethanol, and dried again at 60°C for 5 minutes. Hydrogen peroxide was added for 10 minutes at RT before washing in distilled water. Target retrieval was performed using the manual method (98–102°C), for 15 min, washed in distilled water, and dehydrated in 100% ethanol and allowed to dry completely at RT. Protease Plus was added to the sections and incubated in the HybEZ II Oven at 40°C for 30 minutes. After washing in distilled water, the sections were incubated with respective probes for 2 hours at 40°C. Probes were detected with Opal 520 or 620 Reagent Pack (FP1487001KT, FP1495001KT; Akoya Biosciences, Marlborough, MA, U.S.A) and DAPI was applied for 30 seconds before mounting. To reduce autofluorescence, slides were incubated with SudanBlack for 5 minutes at RT. Images were captured using a Nikon C1 confocal microscope (×60 objective, oil) at a resolution of 1024 × 1024 px.

### Statistical Analysis

Gene expression fold-change data (2^−∆∆Cq^) for each target gene was analyzed using Kruskal–Wallis with Dunn’s post hoc multiple comparison test to compare groups within the normal aging, disease group, and Braak tangle stage cohorts. Simple linear regression was used to fit lines of best fit between 2 data sets, with the coefficient of determination (*r*^2^) to express the goodness of fit. Spearman’s rank correlation was used to compare gene expression data and linear variables. All statistical analyses were performed using GraphPad PRISM.

## Results

### Classical RAS Gene Expression Is Elevated in the Midfrontal Cortex in Normal Aging

**Figure 1.**
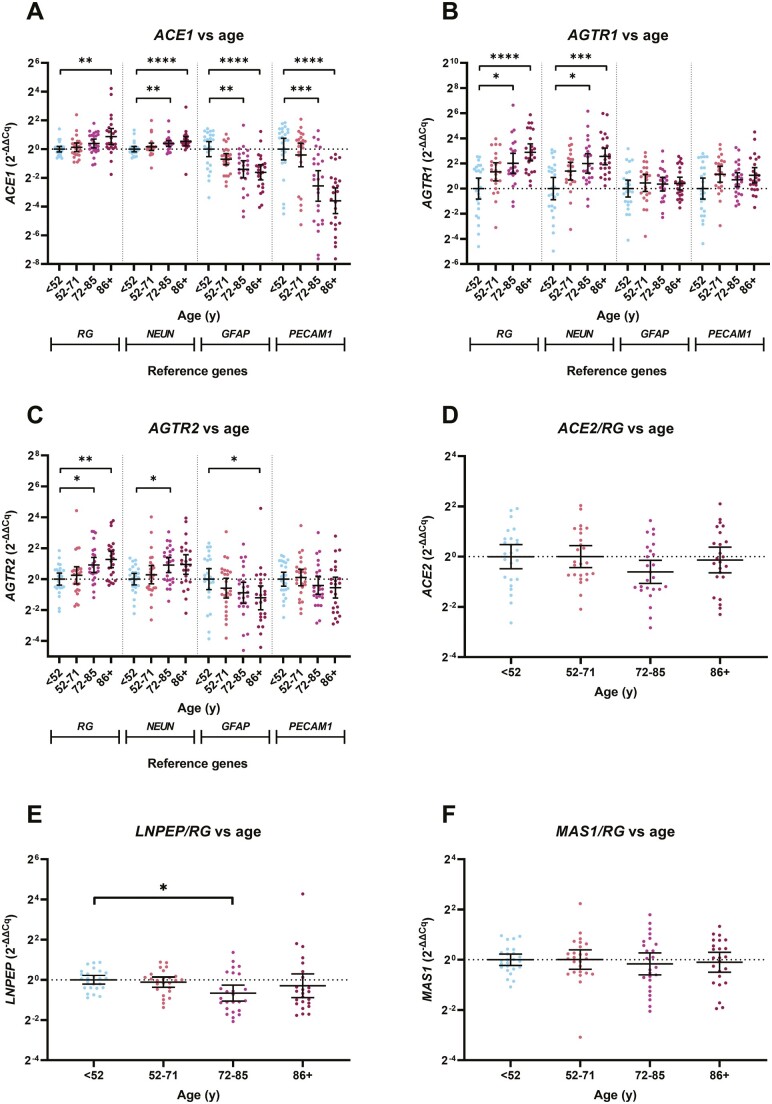
RAS gene expression in the frontal cortex in normal aging. *ACE1*, *AGTR1*, and *AGTR2* gene expression was measured by qPCR in a normal aging cohort (*n* = 99) split into quartiles: <52 years (*n* = 25), 55–71 (*n* = 25), 72–85 (*n* = 25), and 86 years and older (*n* = 24). Genes were calibrated to either reference genes or cell-specific markers (where appropriate) and expressed using the 2^−∆∆Cq^ method. Each dot represents an individual case measured in triplicate. The geometric mean and 95% confidence interval are shown. *p < .05, **p < .01, ***p < .001, ****p < .0001. RAS = renin–angiotensin system.

*ACE1* and *AGTR1* gene expression, calibrated to reference genes (RG), was significantly increased in relation to age within the frontal cortex in the normal aging cohort (Figure 1A and B). *ACE1/RG* was highest in the 86+-year-old group and was significantly higher compared to the <52-year-old group (*p* < .05; Figure 1A). *AGTR1/RG* was elevated in both the 72- to 85-year-old group (*p* < .05) and the 86+ year-old group (*p* < .01) compared to the <52-year-old group (Figure 1B). Both *ACE1/RG* and *AGTR1/RG* expression correlated positively with age across the entire aging cohort (Spearman’s correlation, *p* = .0001 and *p* < .0001, respectively; Supplementary Figure 2A and B). *ACE1/RG* and *AGTR1/RG* expression were unaffected by gender, hypertensive status, or use of RAS-targeting medication (Supplementary Figures 3–5).

**Figure 2.**
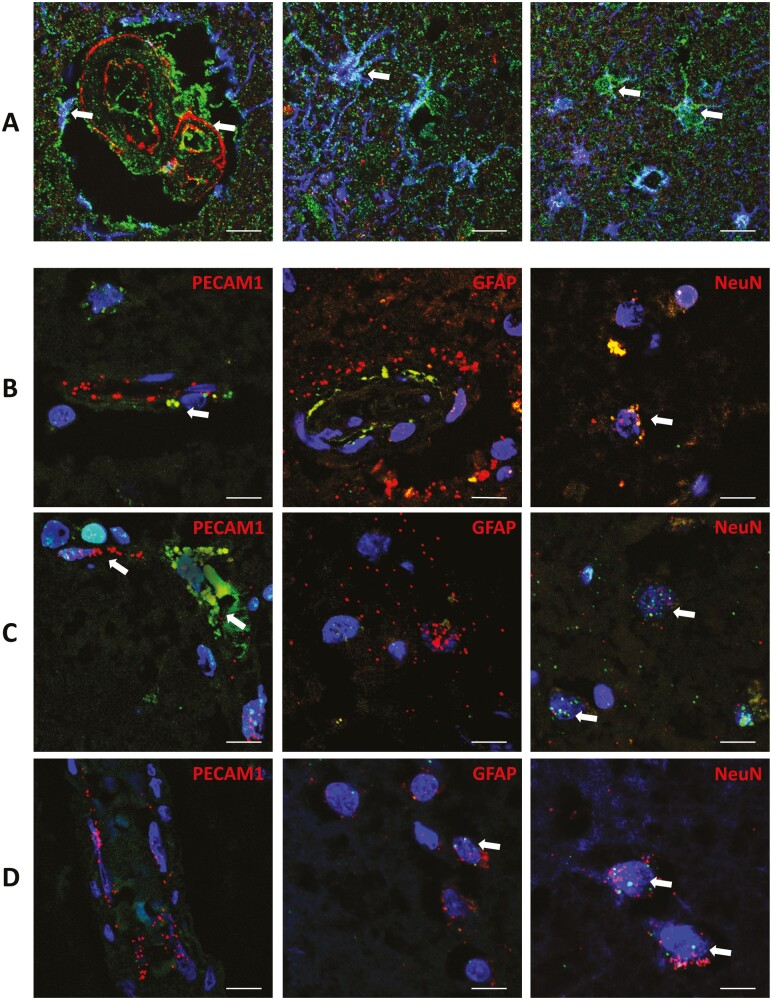
Laser-scanning confocal imaging of the cell-specific expression of ACE-1 (ACE) and ACE-2 proteins, and *AGTR1*, *AGTR2*, and *MAS1* gene expression in the frontal cortex. (A) Representative images of immunofluorescence labeling of ACE-1 (red), ACE-2 (green), and GFAP (blue) in frontal cortex showing vascular and perivascular expression of ACE-1 and ACE-2 (left), ACE-2 expression in astrocytes (center), and likely neuronal expression of ACE-2 in GFAP-negative cells with neuronal morphology (right). (B–D) Representative RNAscope images of cell-specific expression of (B) *AGTR1*, (C) *AGTR2*, (D) *MAS1* transcripts (green) in the frontal cortex, co-labeled with *PECAM1* (left), *GFAP* (center), and *NeuN* (right) probes (red) and DAPI (blue). Scale bars = 10 µm. ACE = angiotensin-converting enzyme.

To explore cell-specific changes in *ACE1* and *AGTR1* gene expression, we used the 2^−∆∆Cq^ method to calculate gene expression relative to the expression of cell-specific markers. To validate the use of the cell-specific markers used in this study, we mapped the expression of ACE-1 and ACE-2 protein by immunofluorescence (IF), and *AGTR1*, *AGTR2*, and *MAS1* genes using immunofluorescence in situ hybridizations (RNAscope), respectively, in FFPE brain tissue sections from the frontal cortex of a nondiseased elderly control donor (Figure 2 and Supplementary Figures 6 and 7). ACE-2 protein was detected in GFAP-labeled astrocyte cell bodies and astrocytic end feet surrounding blood vessels, in addition to GFAP-negative cells likely to be neurons. ACE-1 protein was detected surrounding the outside of arterioles. *AGTR1* and *AGTR2* gene expression was detected in the *PECAM1*-labeled vasculature, *MAS1* gene expression was identified in *GFAP*-labeled astrocytes, and all 3 genes were expressed in neurons labeled with *NeuN*.

The relative gene expression of cell-specific markers: *GFAP* (astrocytic), *NEUN* (neuronal), and *PECAM1* (vascular), in relation to age, is shown in Supplementary Figure 8. *NEUN* gene expression was unchanged, whereas *GFAP* and *PECAM1* were both significantly elevated in relation to age: *GFAP* and *PECAM1* gene expression were higher in the 72- to 85-year age group (*p* < .001), and the +86-year-old age group (*p* < .0001), compared to the <52-year age group.

*ACE1* and *AGTR1* gene expression were both increased with age when calibrated to the neuronal marker, *NEUN*, indicating increased neuronal expression (Figure 1A and B). The relative expression of astrocytic (*ACE1/GFAP*) and endothelial (*ACE1/PECAM1*) *ACE1* was reduced with normal aging. In contrast, *AGTR1/GFAP* and *AGTR1/PECAM1* expression were unaltered across the different age groups (Figure 1B).

### AGTR2 Gene Expression Is Elevated in Normal Aging

*AGTR2/RG* gene expression was increased in relation to age and was significantly higher in the 72- to 85-year-old (*p* < .05) and 86+year-old (*p* < .01) compared to the <52-year age group (Figure 1C)*. AGTR2/RG* expression correlated positively with age across the whole cohort (Spearman’s, *p* < .0001; Supplementary Figure 2C). When calibrated to cell-specific markers, *AGTR2/NEUN* was increased indicating elevated neuronal expression*. AGTR2/GFAP* was reduced, indicative of reduced astrocytic *AGTR2* expression, and was unchanged when calibrated to *PECAM1* (Figure 1C). *AGTR2/RG* expression was unaffected by gender or hypertensive status, or use of RAS-targeting medication (Supplementary Figures 3–5).

### ACE2 and MAS1 Gene Expression Were Unchanged, Whereas LNPEP Expression Is Reduced in the Midfrontal Cortex in Relation to Normal Aging

*ACE2/RG* and *MAS1/RG* gene expression were unaltered in the frontal cortex in relation to age (Figure 1D–F) and did not correlate with age across the entire cohort (Supplementary Figure 2D and 2F). *LNPEP* expression was, however, significantly lower in 72- to 85-year-olds compared to ­<52-year-olds and *LNPEP/RG* expression correlated negatively with age across the entire cohort (*p* = .0025, Supplementary Figure 2E). *ACE2/RG*, *MAS1/RG*, and *LNPEP/RG* expression were all unrelated to gender or hypertensive status (Supplementary Figures 3 and 4). In the +86-year-old age group, *ACE2/RG* expression was significantly higher in cases treated with a RAS-targeting medication (Kruskal–Wallis with Dunn’s post hoc test, *p* = .0134; Supplementary Figure 5).

### RAS Gene Expression Is Dysregulated in AD in Relation to Braak Tangle Stage

**Figure 3.**
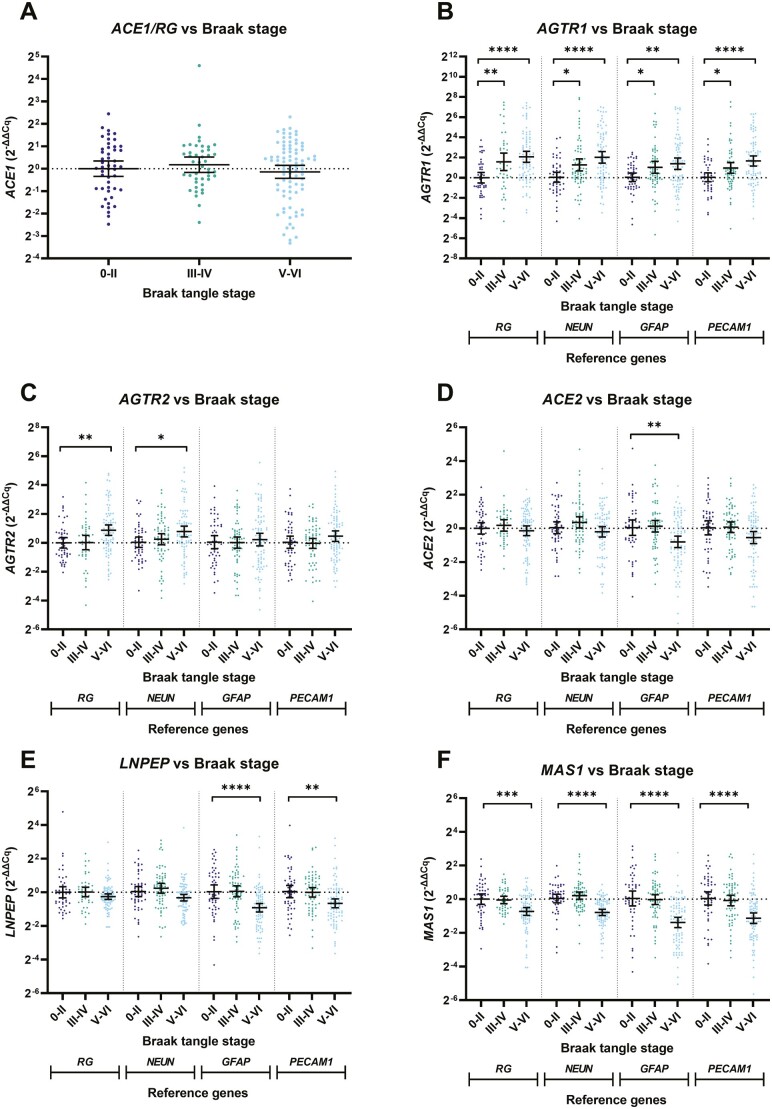
Altered RAS receptor gene expression in the frontal cortex in relation to Braak tangle stage in dementia. *AGTR1* and *AGTR2* gene expression is elevated, whereas *LNPEP* and *MAS1* gene expression is reduced, in relation to Braak tangle stage groups: 0–II (*n* = 48), III–IV (*n* = 44), and V–VI (*n* = 85) in a cohort comprising Alzheimer’s disease, mixed (AD/VAD), and age-matched controls. Gene expression was measured by qPCR and calibrated to reference genes and cell-specific markers where appropriate. Relative gene levels are expressed using the 2^−∆∆Cq^ method. Each dot represents an individual case measured in triplicate. The geometric mean and 95% confidence interval are shown. *p < .05, **p < .01, ***p < .001, ****p < .0001. RAS = renin–angiotensin system.

We next investigated whether RAS gene expression was altered in AD in relation to Braak tangle stage (BS), which is a pseudo-temporal marker of disease stage (23). The control, AD, and mixed AD/VaD cohort were combined and stratified: BS0–II (*n* = 48), BSIII–IV (*n* = 44), and BSV–VI (*n* = 85). *ACE1/RG* expression was unchanged in relation to BS (Figure 3A). In contrast, *AGTR1/RGs* was increased at BSIII–IV (*p* < .05) and BSV–VI (*p* < .01) compared to BS0–II (Figure 3B).

We next calculated the relative changes in *ACE1* and *AGTR1* adjusted for cell-specific markers. The gene expression of cell-specific markers in relation to BS is shown in Supplementary Figure 9. *AGTR1/NEUN* was elevated in relation to BS likely reflecting increased neuronal *AGTR1* expression (Figure 3B) with disease progression. *AGTR1/GFAP* and *AGTR1/PECAM1* gene expression were also increased in relation to BS (Figure 3B).

*AGTR2/RG* gene expression was increased in BSV–VI compared to BS0–II (*p* < .01; Figure 3C). A relative increase in *AGTR2/NEUN* indicates higher neuronal expression (as observed for *AGTR1*; Figure 3C). In contrast, *AGTR2/GFAP* and *AGTR2/PECAM1* were unaltered in relation to BS (Figure 3C) indicating that elevated *AGTR2* was possibly due to increased astrocyte and blood vessel content.

*ACE2/RG* expression was unaltered in relation to BS (Figure 3D). A trend toward lower *LNPEP* in BSV–VI was observed, which approached significance after adjustment for *NEUN* (*LNPEP/NEUN*, Dunn’s *p* = .0747), and was significant when adjusted for *GFAP* (p < .0001) and *PECAM1* (p < .01) (Figure 3E).

*MAS1/RG* expression was highly significantly reduced in BSV–VI compared to BS0–II (*p* < .001) and was lower in BSV–VI when calibrated against all 3 cell-surface markers (p < .0001 for all) indicating a global reduction in *MAS1* gene expression in end-stage AD (Figure 3F).

We determined if RAS gene expression was related to parenchymal Aβ or tau load within the frontal cortex (Supplementary Figures 10 and 11). *AGTR1/RG* and *AGTR2/RG* correlated positively with tau load (*r* = 0.24, *p* = .004 and *r* = 0.27, *p* = .001, respectively) and *MAS1/RG* correlated inversely with parenchymal Aβ load (*r* = −0.21, *p* = .012) and tau load (*r* = −0.29, *p* = .0003). No other RAS genes correlated with Aβ or tau load.

### RAS Gene Expression Is Differentially Regulated in AD Compared to VaD

**Figure 4.**
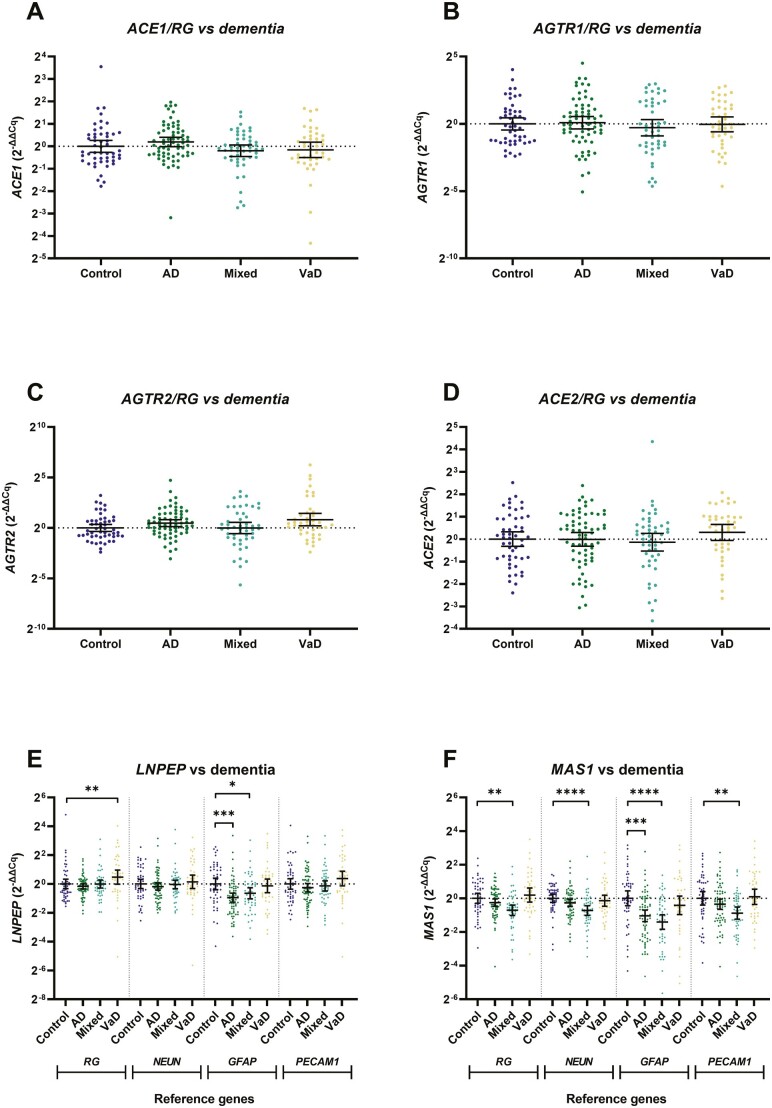
*ACE1*, *ACE2*, *AGTR1*, and *AGTR2* gene expression is unaltered but *LNPEP* and *MAS1* gene is dysregulated in the frontal cortex in dementia. Gene expression was measured by qPCR in a cohort (*n* = 209) divided into diagnoses group: age-matched controls (*n* = 51), Alzheimer’s disease (AD; *n* = 66), AD and VaD (mixed; *n* = 50), and vascular dementia (VaD; *n* = 42). Genes were calibrated to reference genes and expressed as using the 2^−∆∆Cq^ method. Each dot represents an individual case measured in triplicate. The geometric mean and 95% confidence interval are shown. *p < .05, **p < .01, ***p < .001, ****p < .0001.

We next compared gene expression in the AD and mixed AD/VaD compared to the VaD group, to determine if changes in RAS gene expression were specific to AD. The gene expression of mediators of cRAS signaling, *ACE1* and *AGTR1*, and *AGTR2*, calibrated to RGs, was unaltered in all 3 dementia subtypes (Figure 4A–C). *ACE2*/*RG* expression was also unchanged between dementia groups (Figure 4D). However, *MAS1*/*RG* was reduced in mixed dementia, compared to controls (*p* < .01; Figure 4F). A similar pattern was observed after adjustment for all 3 cell-specific marker genes indicating a widespread reduction in *MAS1* gene expression across all cell types (Figure 4F); the expression of cell-specific marker genes across the dementia groups is shown in Supplementary Figure 12).

*LNPEP1*/*RG* was increased in VaD compared to age-matched controls (p < .01; Figure 4E). *PECAM1* expression remained unchanged in VaD, suggesting that blood vessels expressed relatively more *LNPEP* in VaD.

RAS gene expression in the case–control cohort was unaltered in relation to gender, hypertensive status, or RAS medication history (Supplementary Figures 13–15) except for *ACE2/RG* gene expression, which was significantly elevated in AD donors prescribed RAS-targeting medication (Dunn’s, *p* = .0495; Supplementary Figure 15D).

## Discussion

In this study, we have compared the gene expression of mediators of classical (*ACE1*, *AGTR1*, *AGTR2*) and regulatory RAS signaling (*ACE2*, *MAS1*, *LNPEP*) in the frontal cortex in normal aging and AD, in relation to disease stage, and compared these changes to VaD. In normal aging, *ACE1* and *AGTR1* gene expression was elevated, whereas *ACE2* gene expression and the counter-regulatory RAS receptors, *MAS1* and *LNPEP*, remained unchanged. *AGTR2* was elevated in normal aging perhaps reflecting a compensatory response to overactive ang-II/AGTR1 signaling. In AD, *AGTR1* gene expression was elevated in BSIII–IV, that is, at an intermediate disease stage, and *AGTR2* gene expression was raised in BSV–VI (possibly in response to cRAS signaling). *MAS1* receptor gene expression, a major downstream rRAS receptor, was reduced at BSV–VI in AD, and in mixed dementia. Dysregulation of RAS gene expression in mixed and pure AD cases was not observed in VaD, with the exception of *LNPEP*, which encodes IRAP, which was elevated. This study, together with our recent biochemical assessment of RAS proteins and enzymes in normal aging and AD (15), indicates that RAS signaling is altered in normal aging but becomes differentially dysregulated in AD.

Age-related increased cRAS signaling in the heart and kidney predispose elderly individuals to chronic ­hypertensive-related diseases (reviewed (24)). Our previous study reported higher ACE-1 and ang-II levels in the frontal cortex in normal aging (15). Here, *ACE1* and *AGTR1* gene expression were also both elevated in relation to age in the frontal cortex providing further evidence for overactivation of cRAS signaling in normal aging. We previously reported that ACE-1 enzyme activity was inversely correlated to elevated ang-II level in normal aging, perhaps due to the ang-II peptide being able to downregulate ACE-1 enzyme activity as part of a protective mechanism against age-related cRAS signaling (25). In contrast, *ACE2* and *MAS1* gene expression were unaltered with age; however, *AGTR2* was increased, possibly as a compensatory mechanism in response to overactive cRAS signaling and *LNPEP* gene expression was reduced. The significance of these age-related changes in cRAS and rRAS pathways warrants further exploration.

RAS signaling was also dysregulated in AD with evidence that the ACE-1/ang-II/AT1R cRAS pathway was overactivated in AD. Savaskan et al., in 2001 reported increased ACE-1, AT1R, and ang-II expression within pyramidal cortical neurons and surrounding cortical blood vessels in AD (9). AT1R protein levels are increased in the hippocampus (26) and elevated AT1R protein levels are related to markers of intracellular signaling and oxidative stress in AD (14). Most studies have focused on end-stage AD; here, we stratified the AD, mixed AD, and age-matched controls, according to Braak tangle stage and found that *AGTR1* gene expression was elevated in BSIII–IV, at an intermediate disease stage. These findings reflect our recent study showing elevated ACE-1 enzyme activity in BSIII–IV in AD (15). A previous study has also shown elevated *AGTR1* in relation to disease progression (27) (although other studies report no change (14)). Together, these data provide further evidence for the overactivation of ACE-1/ang-II/AT1R signaling, possibly in the early stages of AD.

*AGTR2* expression was increased in AD at BSV–VI, representing end-stage disease. The number of AT2R binding sites, revealed by radiolabeled ang-II binding, are elevated in the temporal cortex in AD (28); however, *AGTR2* gene expression has also been reported to be lower in AD (14). *AGTR2* is upregulated in other disease states associated with overactive cRAS, possibly to limit the damage caused by chronic *AGTR1* signaling (reviewed (29)). Our data suggest that elevated *AGTR2* expression is likely a result of a compensatory response to counteract the potentially damaging effects of cRAS signaling in both normal aging and AD.

Overactivity within the cRAS axis is associated with downregulation of the counter-regulatory rRAS pathways, including reduced ACE-2/Ang-(1–7)/MasR signaling and dysregulated ang-III/AT4R signaling in AD (11–13). In this study, *MAS1* gene expression was strongly reduced in end-stage disease, at Braak stage V–VI, and in the mixed AD cohort but not in VaD. The timing of altered cRAS and rRAS signaling in AD is unclear (30) but our gene expression data implies that rRAS signaling is dysregulated at end-stage disease following earlier changes in cRAS gene expression, and is therefore likely to be a response (rather than a cause) to overactivation of cRAS signaling. Interestingly, *MAS1* gene expression was inversely correlated with both Aβ (4G8) and tau (AT8) load. This supports our previous study, which showed that reduced ACE-2 activity in AD was inversely correlated with elevated parenchymal Aβ and tau load (13). CNS-administered ang-II dose-dependently stimulates amyloidogenic APP processing and Aβ production (31), and enhances tau phosphorylation (32) in adult Wistar rats, possibly via induction of CDK5 and MAPTK kinases (33). The potential relationship between RAS signaling and tau pathology warrants further investigation.

Gene expression within brain tissue homogenates provides an overall picture of RAS homeostasis in both aging and dementia but does not provide useful information regarding cell-type specific alterations in RAS. To attempt to address this limitation, we have adjusted gene expression using cell-specific gene markers. Our immunofluorescence and in situ hybridizations findings indicate that ACE-1 is primarily localized within blood vessels, as previously reported (12), but is also expressed within neurons and scattered parenchymal astrocytes. ACE-2 was enriched in astrocytes, in the parenchyma, and on astrocytic end feet surrounding blood vessels. ACE-2 also labeled neurons and blood vessels to a lesser degree. These findings support previous studies in human and murine brain tissue (34,35). *MAS1* gene expression was mostly expressed in *NeuN*-labeled neurons and *GFAP*-labeled astrocytes. In rat brain, MAS1 is expressed in neurons and to a lesser extent astrocytes, within the cardiovascular regulatory centers including the hypothalamus, and the hippocampus and cortex (36)—and declines with age (37). Our analysis was not exhaustive, we did not determine if RAS receptors co-localized with microglia, in which RAS receptors have been proposed to regulate microglial phenotype (2) and we were unable to assess *LNPEP* expression in this study.

Adjusting gene expression data using cell-specific markers has potential limitations as it is unclear whether the expression of cell-specific markers used in this study, GFAP (astrocytes) and NeuN (neurons), are altered in relation to changes in cell density or instead reflect changes in intracellular expression. Although studies have shown that GFAP gene and protein expression is increased with aging in rodent and human brain tissue (38,39); recent studies indicate that this does not necessarily reflect a change in astrocytic cell density (27,40–42). A similar cautionary note must also be applied when considering NeuN as a marker of neuronal density. NeuN-labeled neuronal populations vary by age according to brain region in female C57BL/6J mice (43); however, the loss of NeuN immunoreactivity did not correlate with reduced neuronal density in a mouse model of cerebral ischemia (44). Using other techniques, such as in situ hybridization, are required to more precisely map changes in the cell-specific distribution of RAS receptors to confirm our findings in future studies.

RAS signaling is known to be influenced by sex hormones, which may account for gender differences in RAS-related diseases (reviewed (45)). Here, we did not find a gender-specific difference in RAS gene expression in the aging or case–control cohorts. Although not the major focus of this study, we were able to perform secondary analysis to test whether RAS gene expression was also influenced by hypertension status and/or RAS-targeting medication. Despite limitations, hypertension status did not appear to strongly influence RAS gene expression; however, *ACE2* gene expression was higher in the >85-year-old group, and in the AD group, for those hypertensive patients prescribed RAS-targeting drugs. Recent evidence indicates that RAS-targeting medications increase ACE-2 activity and shifts the balance toward rRAS pathways (46).

This postmortem observational study examines gene expression at the time of death and therefore the effect of agonal state, postmortem delay, and RNA quality may affect the interpretation of the data. Alterations in RIN values may be an additional confounder in this study; however, a previous study showed that postmortem delay and pH have only a modest effect on RIN values in postmortem brain tissue (47) and we have found that RIN values were not affected by postmortem delay but were significantly higher in relation to tissue pH, as shown by a previous study (48). Overall, our RIN values were lower than would be ideal but were relatively unaffected by age, in both the aging and case–control cohort, supporting previous findings (47). RIN values were lower in AD compared to age-matched controls, supporting findings from White and colleagues (47). We acknowledge that RIN values may account for some of the disease-related changes in the expression of the cell-specific calibrators (*PECAM1* and *GFAP* but not *NEUN* as this remained stable), or RAS genes; however, RIN values in isolation should be used cautiously as they have been shown to be a poor predictor of cDNA quality in human postmortem brain tissue (49).

In conclusion, we show elevated cRAS gene expression in normal aging: *ACE1*, *AGTR1,* and *AGTR2* gene expression, was elevated, but rRAS genes were unaltered. In AD, *AGTR1* and *AGTR2* genes were elevated in BSIII–IV and BSV–VI, respectively. *MAS1* was globally reduced in AD at BSV–VI, was lower in mixed AD/VaD, and inversely correlated with both Aβ and tau pathology. Overall RAS gene expression in AD and mixed AD/VaD differed from gene expression in pure VaD—suggesting altered RAS signaling is driven by AD-specific pathogenic processes. Together with our recently published study (15), these data provide a deeper understanding of RAS in relation to age and AD and suggest that RAS dysregulation in AD differs from normal aging that can potentially be exploited as future therapeutic targets in AD.

## Supplementary Material

glad241_suppl_Supplementary_Tables_1-3_Figures_1

## Data Availability

All data within the article are linked to the MRC UKBBN by a unique numeric MRC UKBBN identifier (Supplementary Table 1).
