# Supplementary material for: Altered Gene Expression Within the Renin–Angiotensin System in Normal Aging and Dementia
Source: J Gerontol A Biol Sci Med Sci. 2023 Oct 9;79(1):glad241. doi: 10.1093/gerona/glad241 (PMC10733177; doi:10.1093/gerona/glad241)
Supplement: glad241_suppl_Supplementary_Tables_1-3_Figures_1 [file glad241_suppl_supplementary_tables_1-3_figures_1.pdf]

Supplementary Tables and Figures

Supplementary Table 1. List of UK brain bank network (UKBBN) identifier numbers for cases used in this study. The internal brain bank identifier number (*) is given for cases not otherwise recorded on the UKBBN.

South West Dementia Brain Bank					
BBN_4200	BBN_8978	BBN_9331	BBN_9420	BBN_24324	BBN006.29640
BBN_4202	BBN_8989	BBN_9332	BBN_9421	BBN_24325	BBN006.29723
BBN_4204	BBN_9028	BBN_9336	BBN_9422	BBN_24326	BBN006.29894
BBN_4205	BBN_9038	BBN_9338	BBN_9426	BBN_24330	BBN006.29917
BBN_4208	BBN_9043	BBN_9340	BBN_9429	BBN_24332	BBN006.30024
BBN_4214	BBN_9050	BBN_9341	BBN_9432	BBN_24337	BBN006.30106
BBN_4215	BBN_9078	BBN_9343	BBN_9433	BBN_24561	BBN006.30165
BBN_4216	BBN_9108	BBN_9344	BBN_9435	BBN_24563	BBN006.30186
BBN_4220	BBN_9123	BBN_9346	BBN_10251	BBN_24564	BBN006.30198
BBN_4223	BBN_9136	BBN_9354	BBN_14398	BBN_24565	BBN006.30842
BBN_4229	BBN_9156	BBN_9359	BBN_14403	BBN_24895	BBN006.30889
BBN_4231	BBN_9164	BBN_9361	BBN_14404	BBN_24896	BBN006.31445
BBN_4232	BBN_9173	BBN_9365	BBN_14405	BBN_24899	BBN006.31488
BBN_4238	BBN_9192	BBN_9367	BBN_14406	BBN_24904	BBN006.31492
BBN_8662	BBN_9198	BBN_9368	BBN_19608	BBN_25025	BBN006.31498
BBN_8669	BBN_9200	BBN_9369	BBN_19613	BBN_26011	BBN006.31516
BBN_8675	BBN_9209	BBN_9371	BBN_19614	BBN_26015	BBN006.32326
BBN_8677	BBN_9217	BBN_9372	BBN_19615	BBN006.26095	BBN006.32326
BBN_8691	BBN_9220	BBN_9377	BBN_19624	BBN006.26096	BBN006.32529
BBN_8700	BBN_9224	BBN_9378	BBN_19626	BBN006.26340	BBN006.32544
BBN_8709	BBN_9242	BBN_9379	BBN_19627	BBN006.26344	BBN006.32578
BBN_8747	BBN_9257	BBN_9387	BBN_19628	BBN006.26345	BBN006.32821
BBN_8770	BBN_9268	BBN_9389	BBN_22622	BBN006.26447	BBN006.32826
BBN_8787	BBN_9274	BBN_9394	BBN_22623	BBN006.26572	BBN006.32845
BBN_8829	BBN_9275	BBN_9395	BBN_22624	BBN006.28766	BBN006.33638
BBN_8835	BBN_9292	BBN_9397	BBN_22625	BBN006.28893	BBN006.33682
BBN_8848	BBN_9293	BBN_9398	BBN_24309	BBN006.29018	BBN006.33692
BBN_8861	BBN_9296	BBN_9401	BBN_24310	BBN006.29470	BBN006.34115
BBN_8919	BBN_9303	BBN_9405	BBN_24311	BBN006.28766	BBN006.34119
BBN_8927	BBN_9308	BBN_9407	BBN_24312	BBN006.28893	BBN006.34151
BBN_8944	BBN_9311	BBN_9409	BBN_24315	BBN006.29018	
BBN_8952	BBN_9313	BBN_9413	BBN_24317	BBN006.29470	
BBN_8968	BBN_9323	BBN_9417	BBN_24319	BBN006.29544	
BBN_8975	BBN_9329	BBN_9419	BBN_24320	BBN006.29614	
Newcastle Brain Tissue Resource					
19920067 *	19940046 *	19960193 *	BBN_7366	BBN003.29753	BBN003.34139
19920144 *	19950157 *	19970041 *	BBN_7609	BBN003.30189	
19920210 *	19960134 *	19980157 *	BBN_13401	BBN003.30191	
19940012 *	19960165 *	BBN_2632	BBN003.26928	BBN003.30805	
Edinburgh Brain and Tissue Bank					
BBN_2360	BBN_2511	BBN_20592	BBN001.28563	BBN001.29533	BBN001.30916
BBN_2486	BBN_2513	BBN_23395	BBN001.28959	BBN001.29693	BBN001.30972
BBN_2487	BBN_2531	BBN_24781	BBN001.29466	BBN001.29824	BBN001.31054
BBN_2495	BBN_2562	BBN001.26124	BBN001.29467	BBN001.29882	BBN001.32548
BBN_2504	BBN_2564	BBN001.26309	BBN001.29525	BBN001.30147	BBN001.33613
BBN_2505	BBN_2572	BBN001.26313	BBN001.29526	BBN001.30169	BBN001.34215
BBN_2506	BBN_14395	BBN001.26797	BBN001.29529	BBN001.30208	
BBN_2510	BBN_15221	BBN001.26976	BBN001.29531	BBN001.30841	

Supplementary Table 2. Details of TaqMan assays for reference genes, cell-specific markers, and target genes for qPCR

Gene name	Type	TaqMan assay ID
UBE2D2	Reference gene	Hs00366152_m1
RPL13	Reference gene	Hs00744303_s1
NEUN	Neuronal cell-type calibrator	Hs01370653_m1
GFAP	Astrocytic cell-type calibrator	Hs00909233_m1
PECAM1	Endothelial cell-type calibrator	Hs01065279_m1
AGTR1	Target gene	Hs00241341_m1
AGTR2	Target gene	Hs02621316_s1
LNPEP	Target gene	Hs00893646_m1
MAS1	Target gene	Hs00267157_s1
REN	Target gene	Hs00982555_m1
ATP6AP2	Target gene	Hs00997145_m1
ACE1	Target gene	Hs01104605_g1
ACE2	Target gene	Hs01085333_m1
AGT	Target gene	Hs00174854_m1

Supplementary Table 3: Details of RAS targets and probes used in this study

RNA target	RNAscope® probe	Cat no.
AGTR1	Hs-AGTR1-No-XMm-C2	526071-C2
AGTR2	Hs-AGTR2	459141
MAS1	Hs-MAS1	548721
PECAM1	Hs-PECAM1-O1-C3	487381-C3
NEUN	Hs-RBFOX3-C3	415591-C3
GFAP	Hs-GFAP-C3	311801-C3
P2ry12	Hs-P2RY12-C3	450391-C3
PDGFRB	Hs-PDGFRB-C4	548991-C4

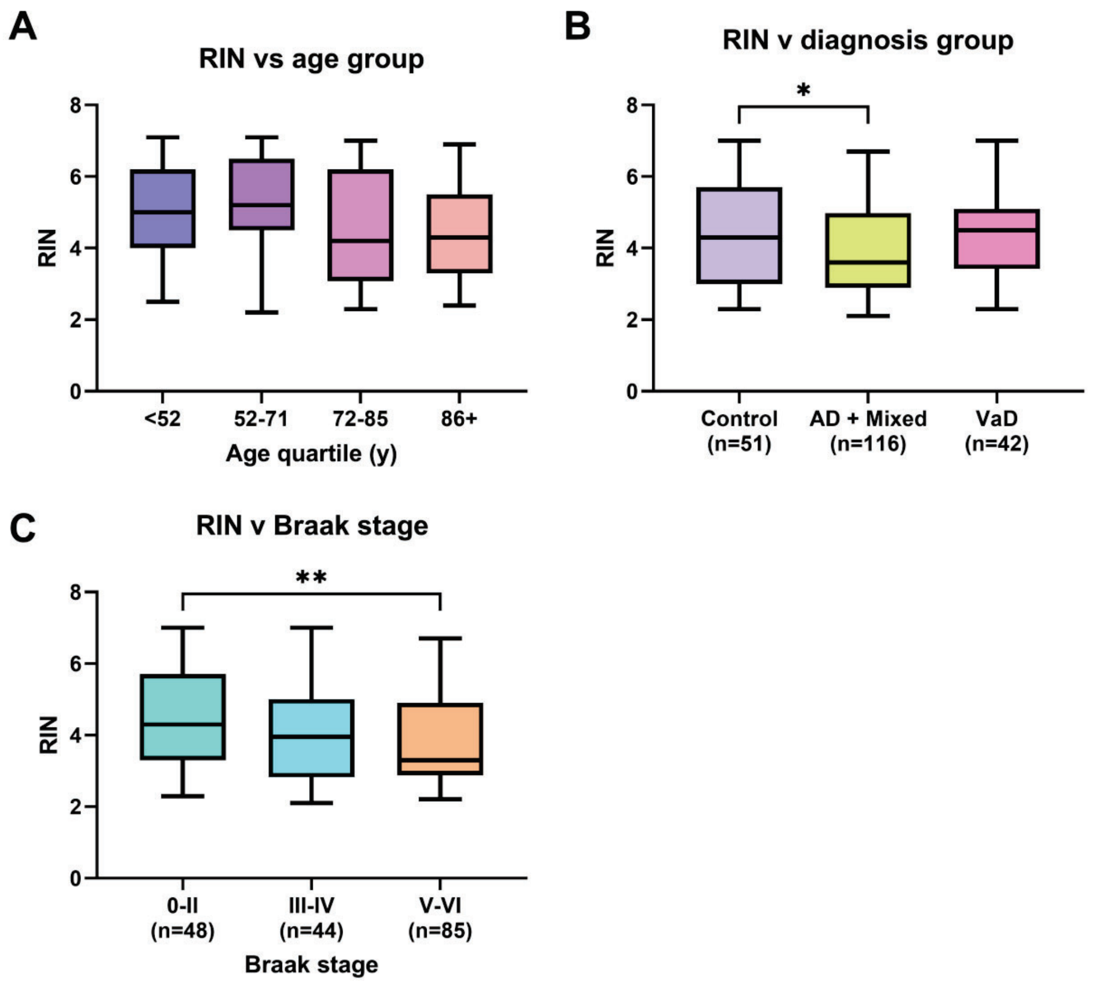

Supplementary Figure 1. Age- and disease-related alterations in RIN number. (**A**) RIN number was not altered in relation to age-at-death in the normal ageing cohort (n = 99). (**B-C**) RIN number was lower in the AD + mixed group (n = 116) compared to age-matched controls (n = 51) and was lower in the Braak tangle stage V-VI group (n = 85) vs the 0-II group (n = 48). * $p < 0.05$, ** $p < 0.01$.

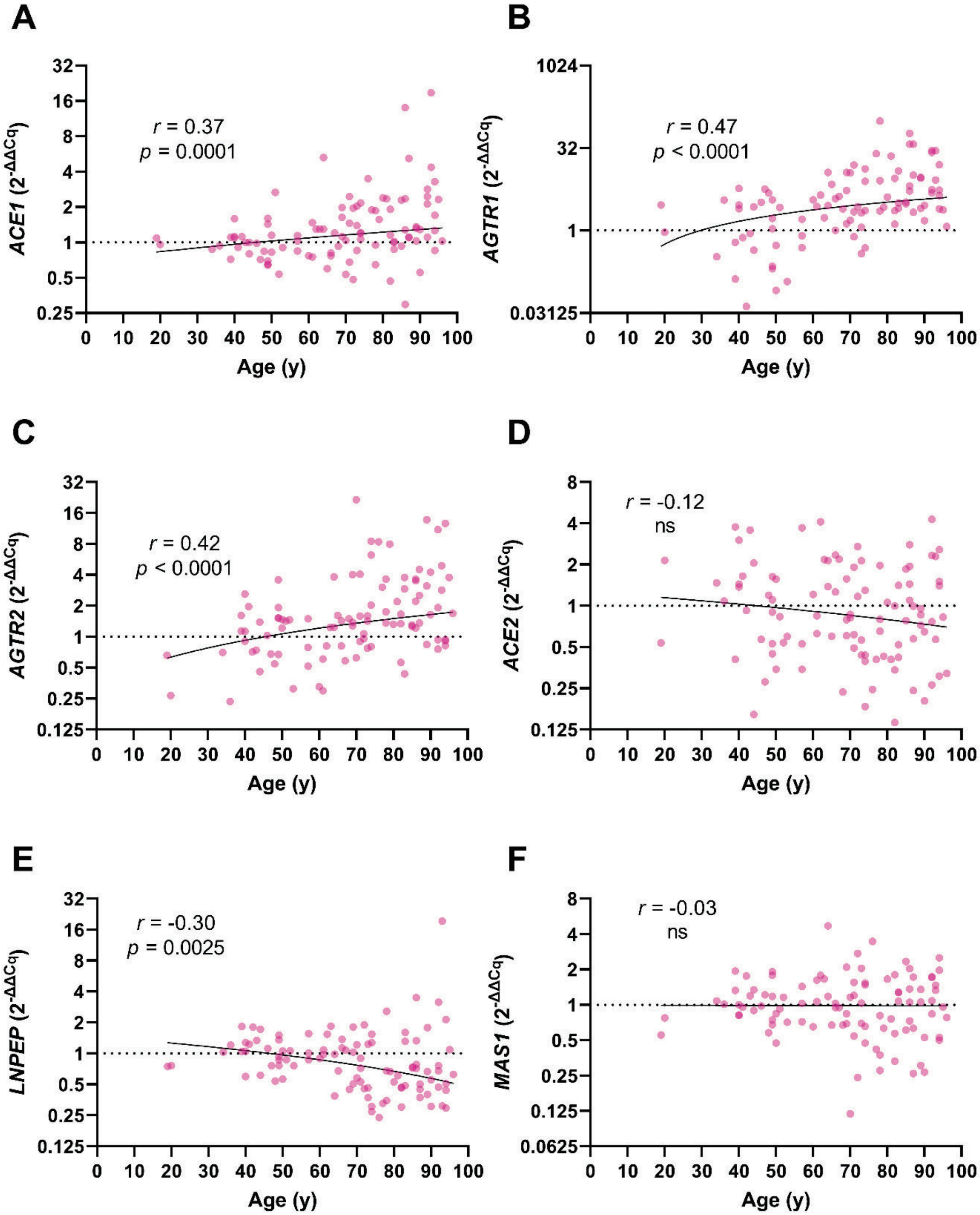

Supplementary Figure 2. Correlation of age and RAS gene expression in the frontal cortex in normal ageing. Gene expression was measured by qPCR in an ageing cohort (n = 99) with respect to reference genes and expressed using the $2^{-\Delta\Delta Cq}$ method. Individual dots represent an individual case measured in triplicate. Spearman's correlation coefficients (*r*), statistical significance (*p* values) and line of best fit (fitted with robust linear regression) are shown.

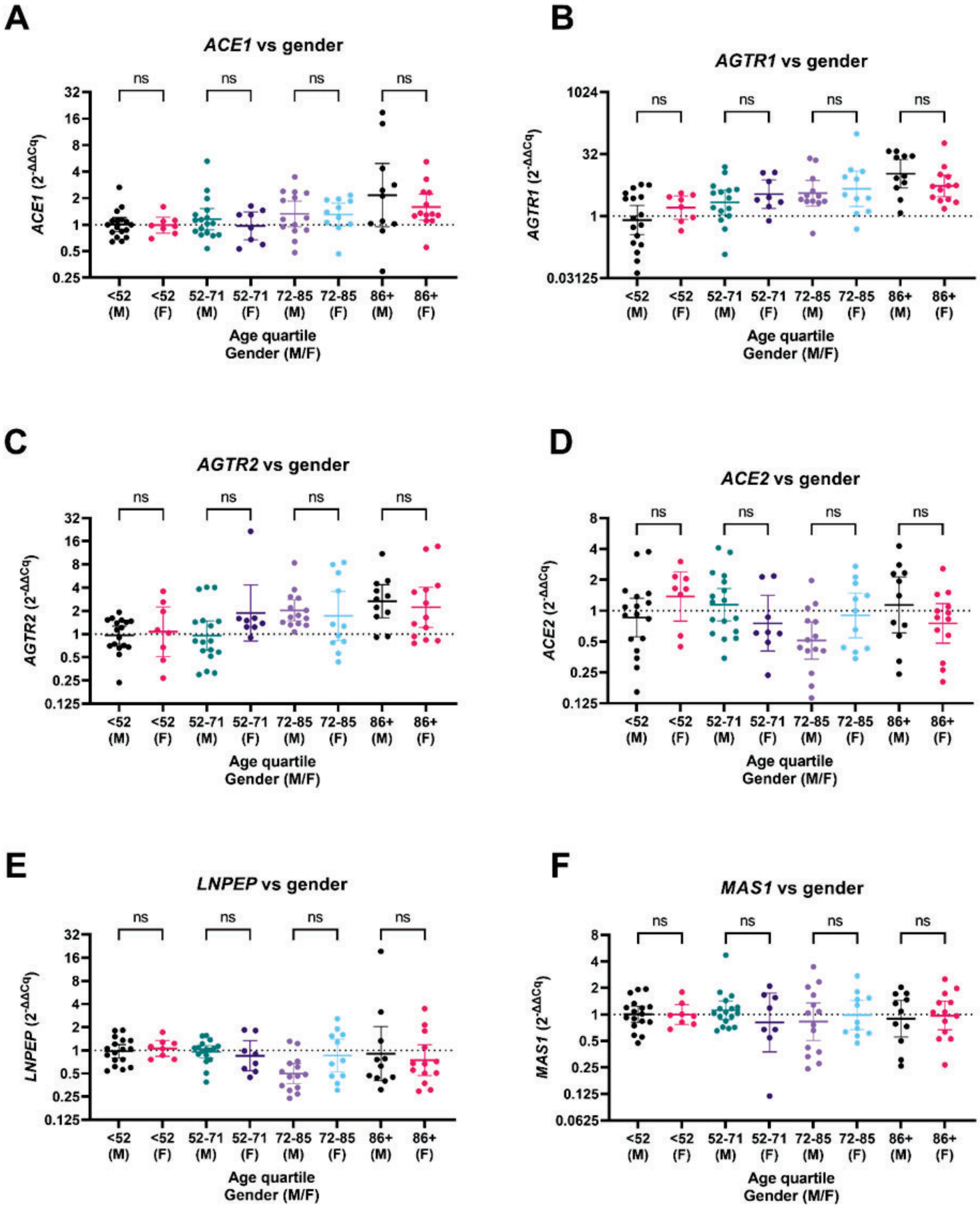

Supplementary Figure 3. Absence of gender-specific alterations in RAS gene expression in normal ageing. No statistically significant effects of gender were observed in the ageing cohort divided into respective age groups: <52 years, 52-71 years, 72-85 years and >85 years. Data were analysed using Kruskal-Wallis with Dunn's posthoc test. Each data point represents gene expression from one individual donor.

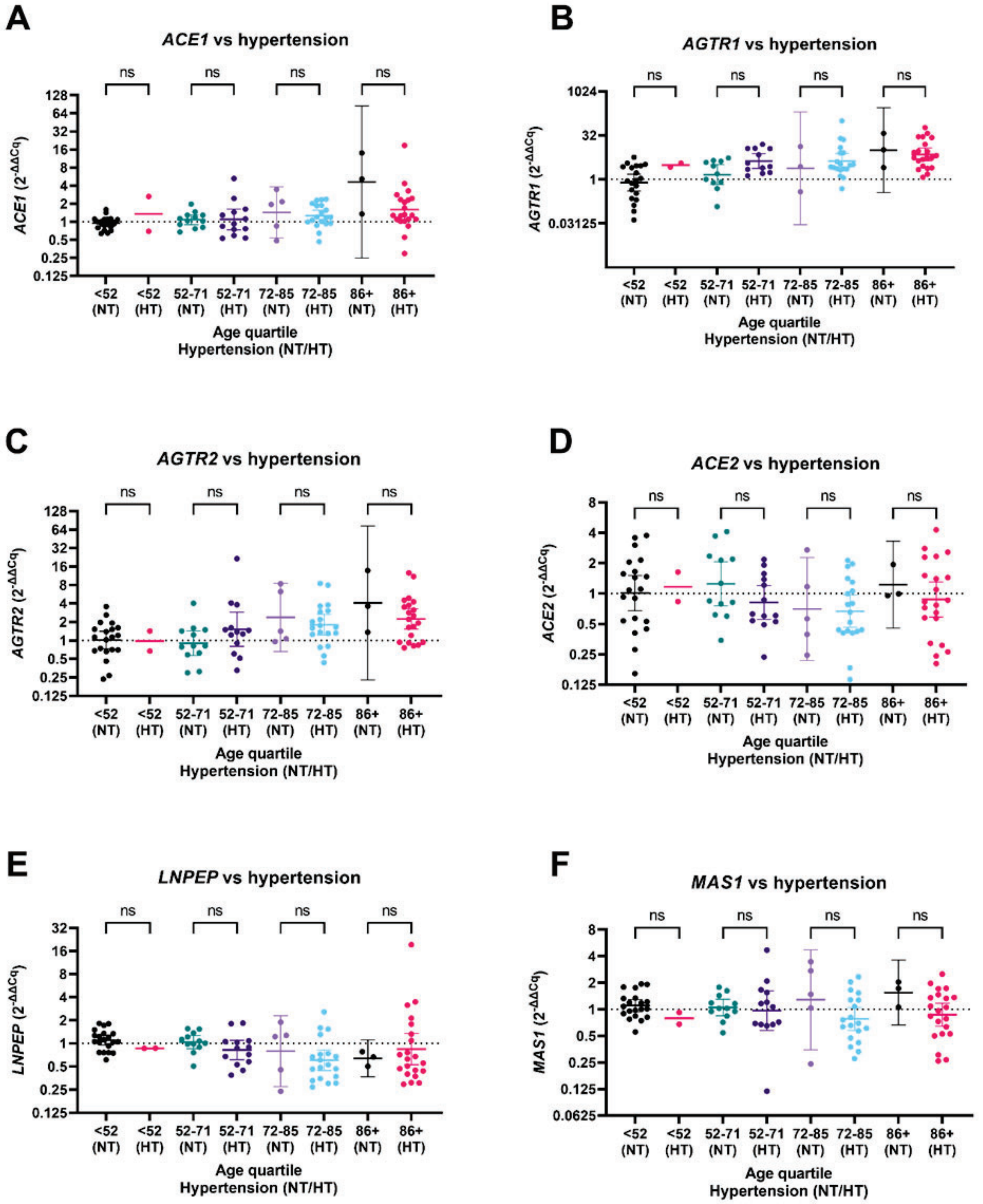

Supplementary Figure 4. Hypertension status is not associated with RAS gene expression in normal ageing in this cohort. No statistically significant effects of hypertensive status on RAS gene expression were observed in the age quartiles. Data were analysed using Kruskal-Wallis with Dunn's posthoc test. Each data point represents gene expression from one individual donor.

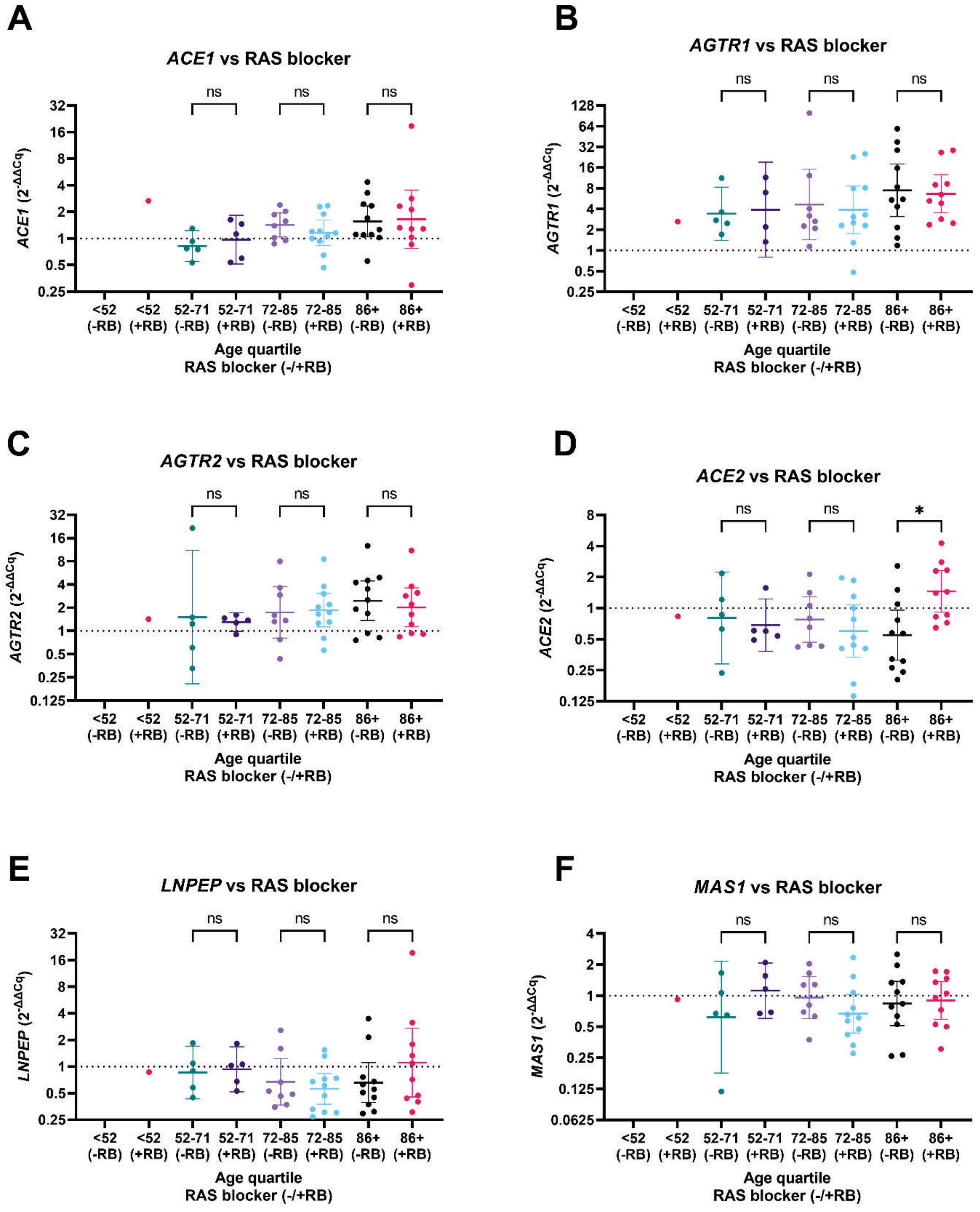

Supplementary Figure 5. A history of RAS medication is associated with elevated ACE2 gene expression in 86+ year-olds. No statistically significant effects of RAS-targeting medication (+RB) on RAS gene expression were observed in the age-group quartiles with the exception of ACE2 gene expression which was higher in 86+ year-olds with a medical history of RAS drug use (Kruskal-Wallis with Dunn's posthoc test; p=0.0134). Data were analysed using Kruskal-Wallis with Dunn's posthoc test. Each data point represents gene expression from one individual donor.

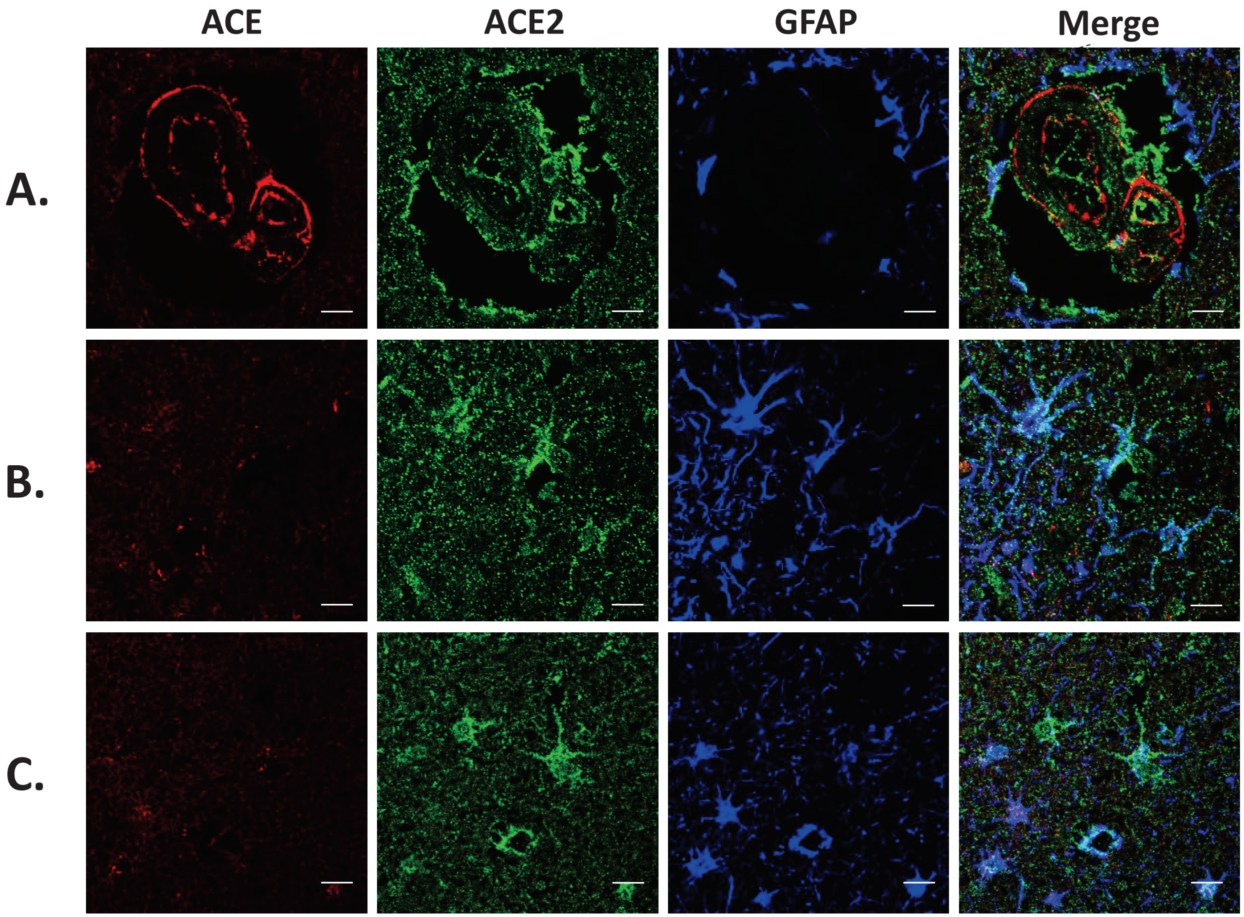

Supplementary Figure 6. LSM imaging of immunofluorescence-labelled ACE-1 (red) and ACE-2 (green) and GFAP (blue) in the frontal cortex showing individual colour channels. Representative images of **A.** vascular and perivascular expression of ACE-1 and ACE-2, **B.** ACE-2 expression in GFAP-labelled astrocytes and **C.** expression of ACE-2 in GFAP-negative cells with neuronal morphology. Scale bars = 10 uM.

A.

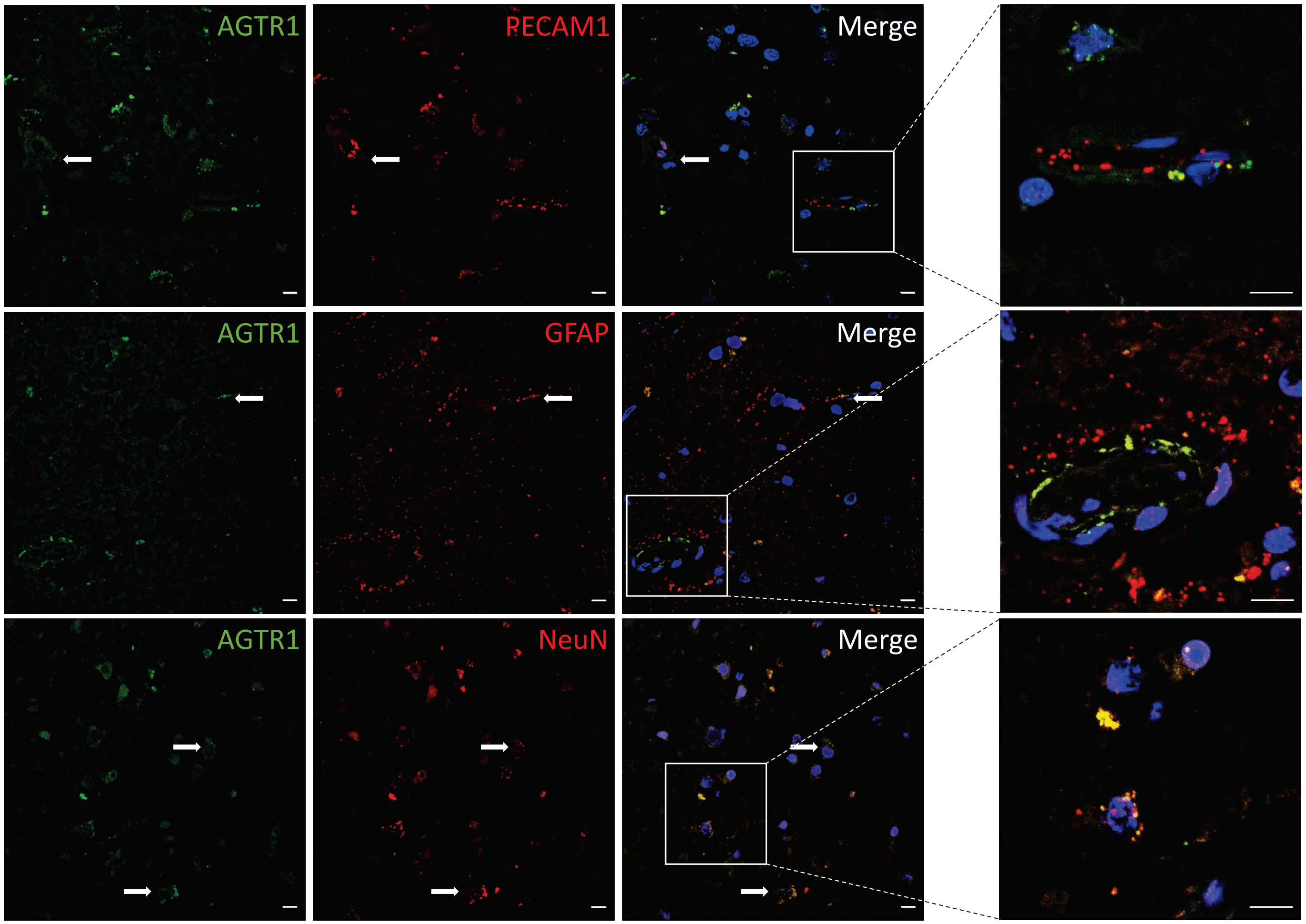

B.

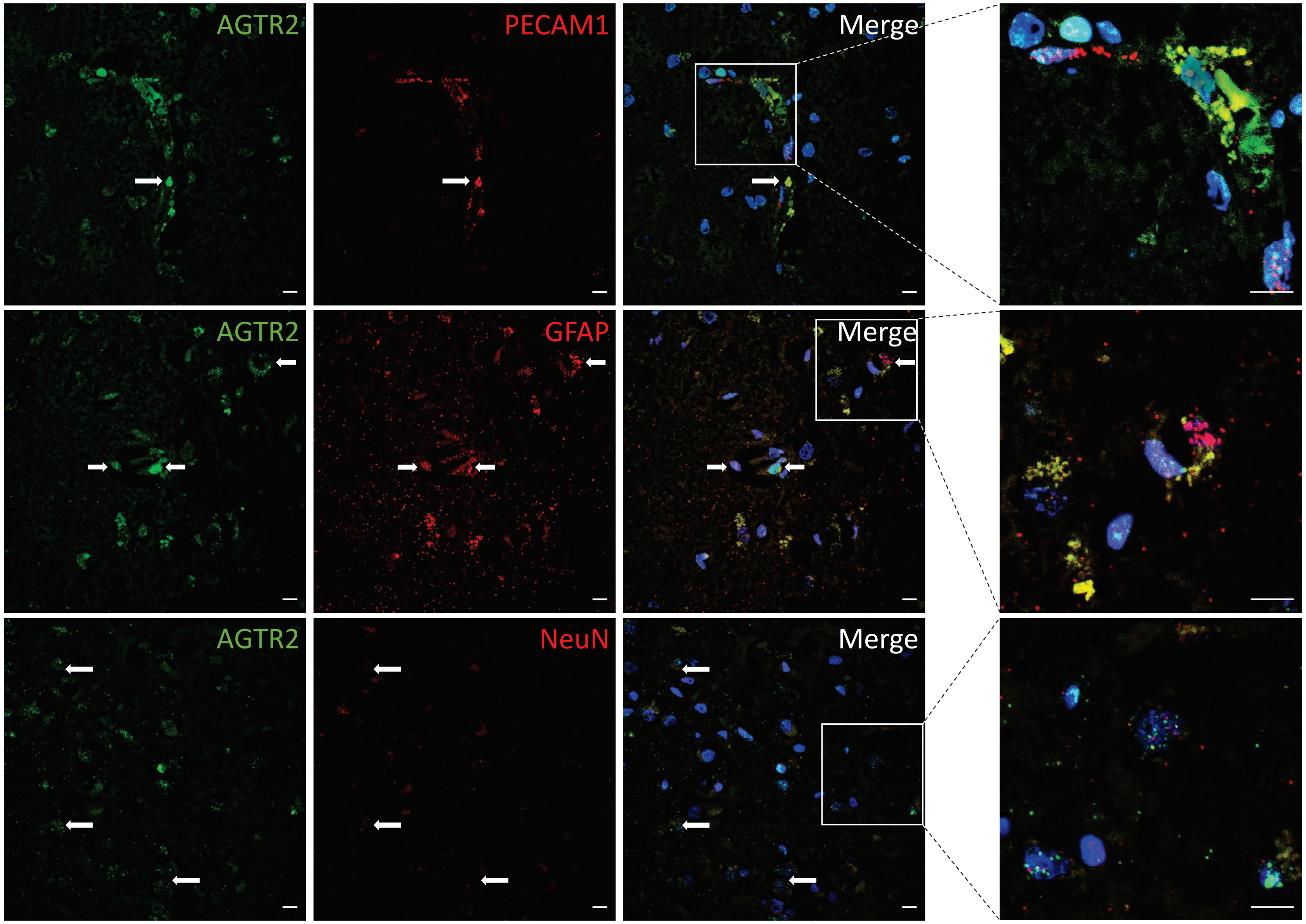

C.

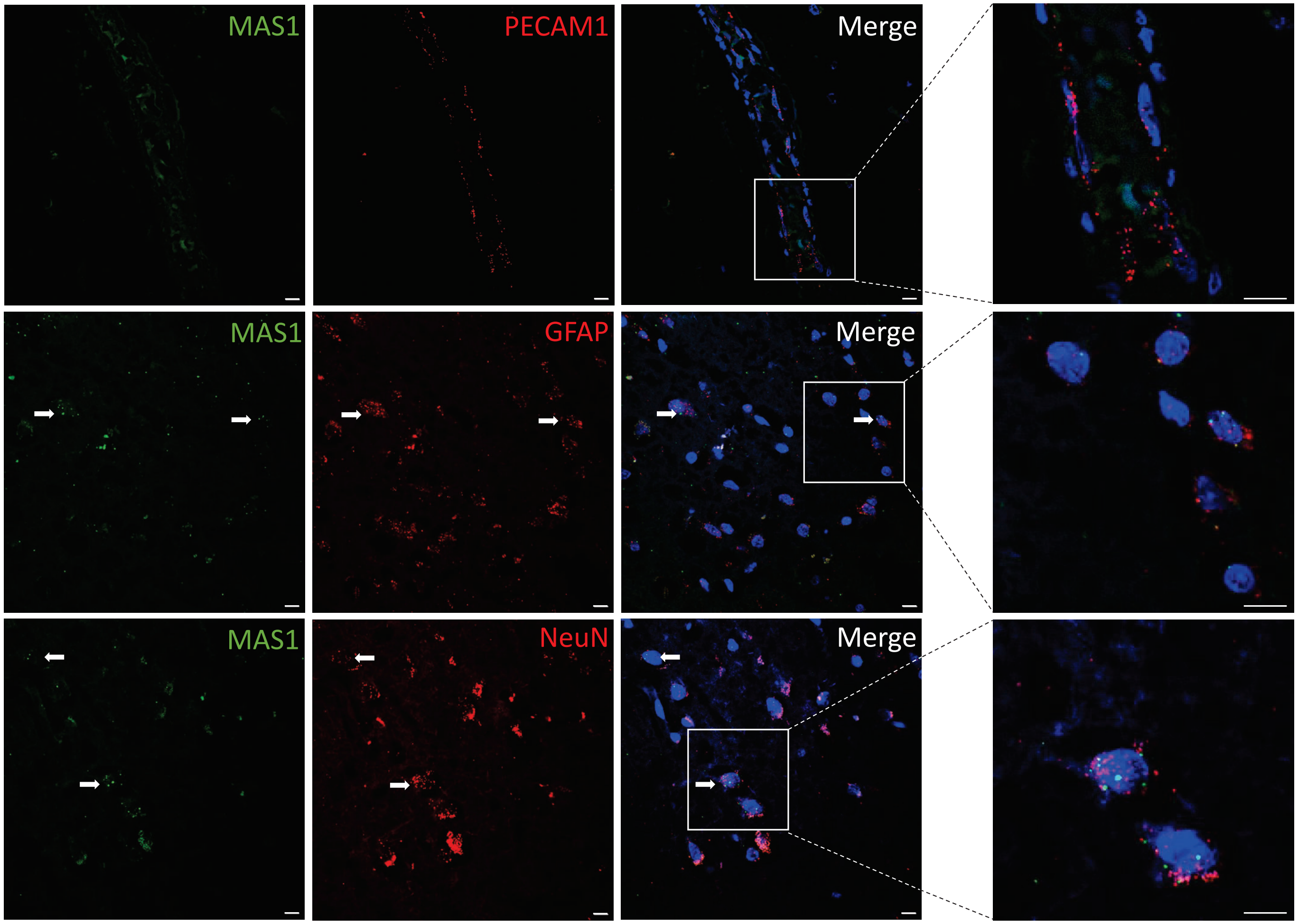

D.

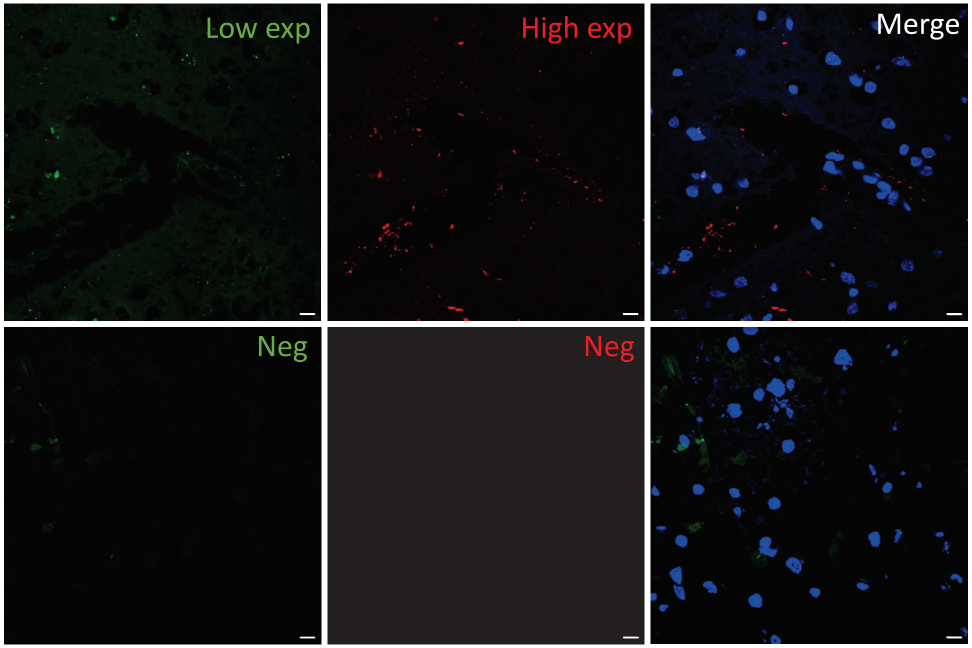

Supplementary Figure 7. LSM imaging of **A.** AGTR1, **B.** AGTR2, and **C.** MAS1 transcripts (green) in the frontal cortex, and **D.** high- and low-expressed positive control probes (red and green) and negative control probes labelled using RNAscope showing individual colour channels. RAS receptor transcripts were co-labelled with **A—C.** cell-specific transcripts (red) PECAM1 (top), GFAP (middle) and NeuN (bottom) and **A—D.** DAPI (blue). Scale bars = 10 µm.

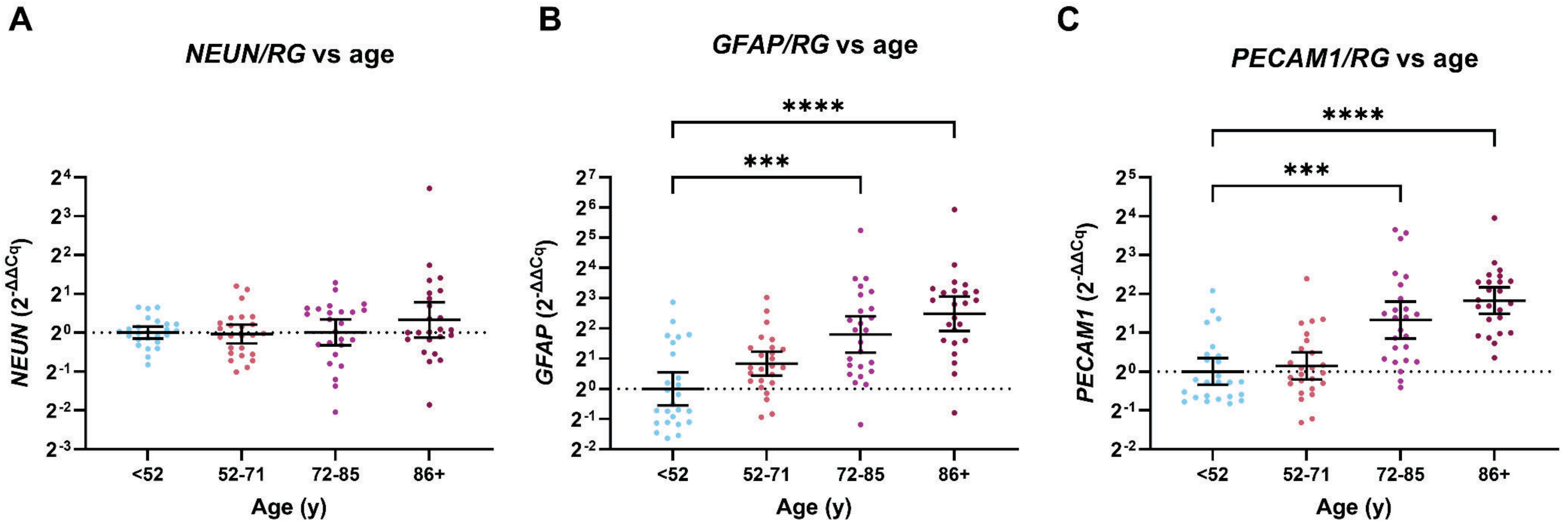

Supplementary Figure 8. Gene expression of cell-specific markers in the frontal cortex in normal ageing. Gene expression was measured by qPCR in an ageing cohort (n = 99) split into quartiles: <52 years (n=25), 52-71 (n = 25), 72-85 (n = 25) and 86 years + (n = 24). Cell-specific gene markers (A. *NEUN*, B. *GFAP*, C. *PECAM1*) were calibrated to reference genes and expressed as using the $2^{-\Delta\Delta Cq}$ method. Individual dots represent an individual case measured in triplicate. The geometric mean and 95% confidence interval are shown. ***p<0.001, ****p<0.0001.

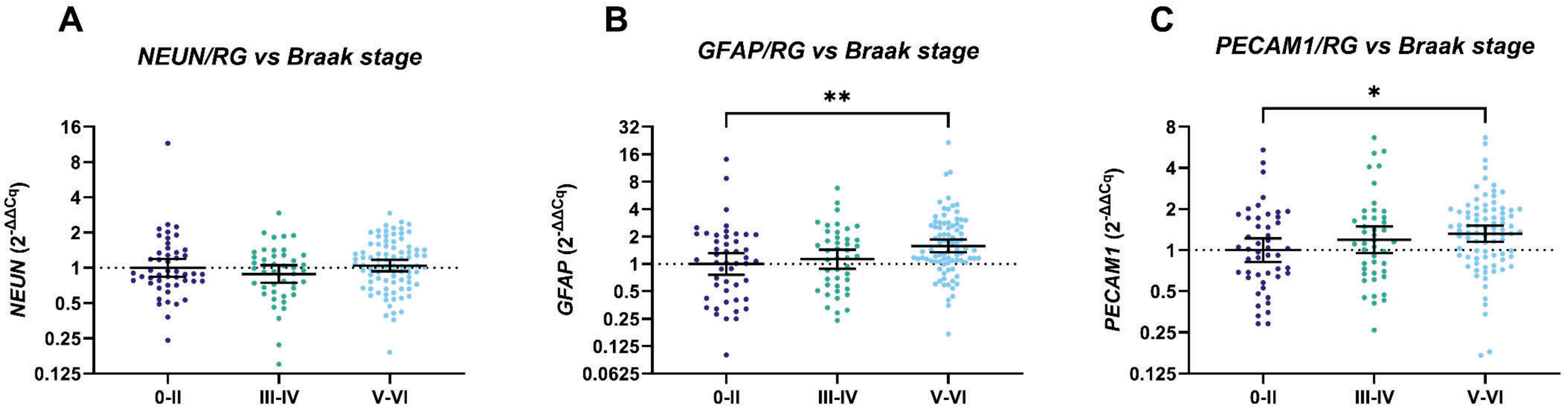

Supplementary Figure 9. Gene expression of cell-specific markers in the frontal cortex in relation to Braak tangle stage. Gene expression was measured by qPCR in the case-control cohort divided into Braak tangle stage groups: 0-II (n=48), III-IV (n=44) and V-VI (n=85). Genes were calibrated to reference genes and cell-specific markers and expressed using the $2^{-\Delta\Delta Cq}$ method. Individual dots represent an individual case measured in duplicate. The geometric mean and 95% confidence intervals are shown. * $p<0.05$, ** $p<0.01$.

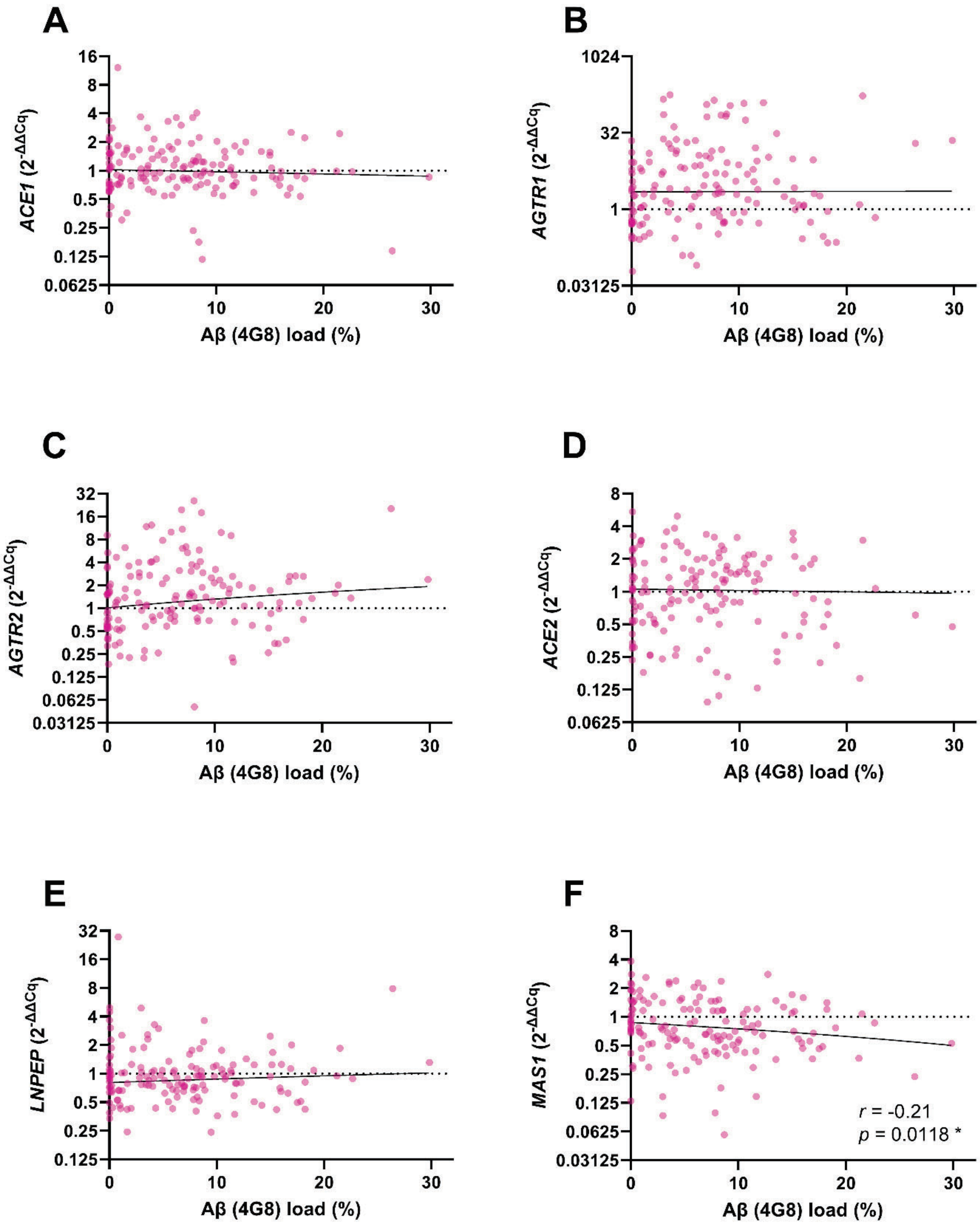

Supplementary Figure 10. No significant correlations between expression of *ACE1, AGTR1, AGTR2, ACE2,* or *LNPEP* and Aβ load in the combined Braak stage cohort. A negative correlation between Aβ load and expression of *MAS1* was significant (Spearman's rank correlation, r = -0.21, p = 0.0118). Line fitted with robust linear regression.

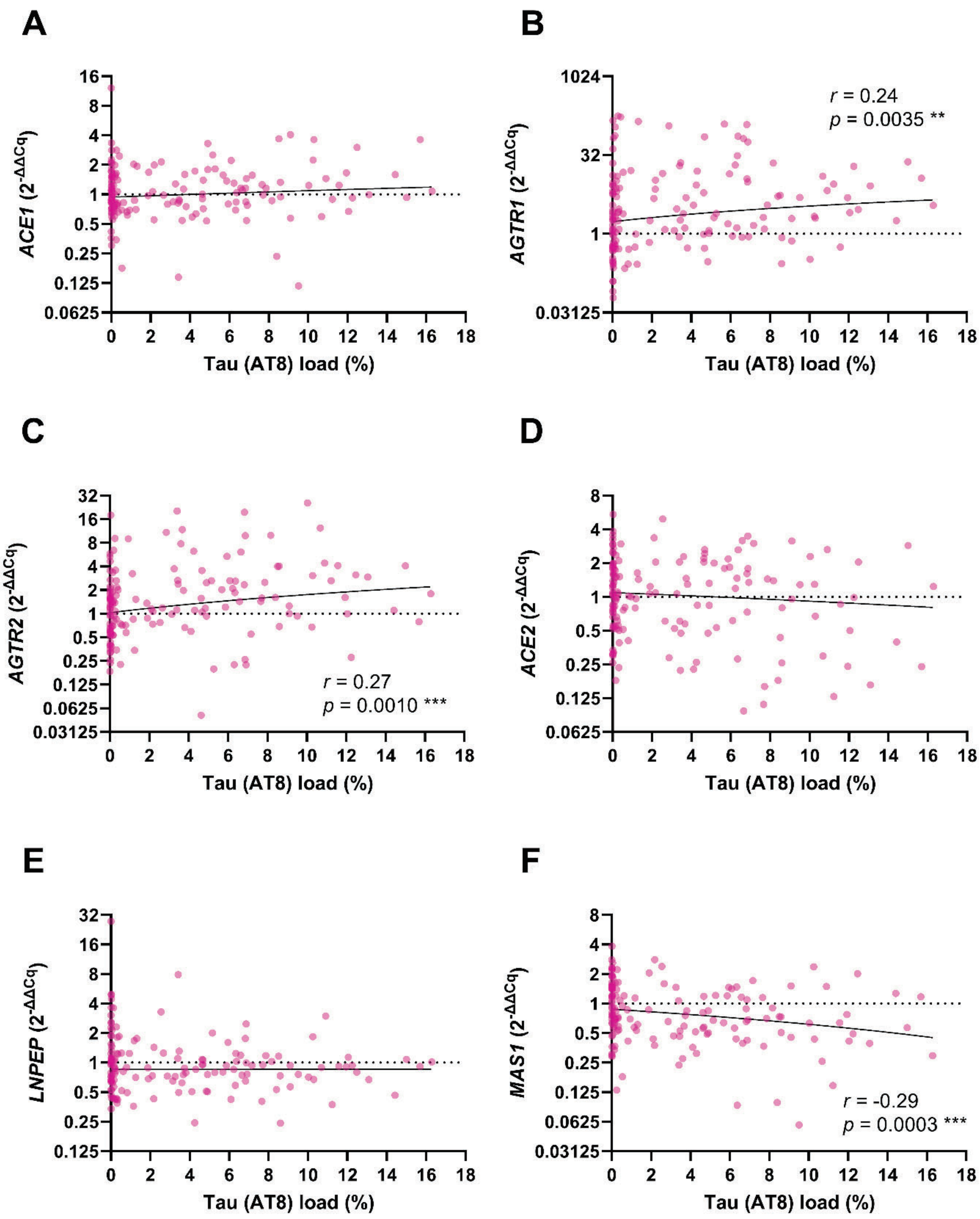

Supplementary Figure 11. *AGTR1*, *AGTR2* and *MAS1* gene expression is related to tau pathology load in the frontal cortex. Positive correlations between Tau load and expression of *AGTR1* and *AGTR2* were significant (Spearman's rank correlation; (**B**) $r = 0.24$, $p = 0.0035$ and (**C**) $r = 0.27$, $p = 0.0010$, respectively). A negative correlation between Tau load and (**F**) *MAS1* expression was also significant (Spearman's; $r = -0.29$, $p = 0.0003$). No significant correlations between Tau load and the expression of *ACE1, ACE2,* or *LNPEP* in the cases from the Braak stage cohort. Lines fitted with robust linear regression.

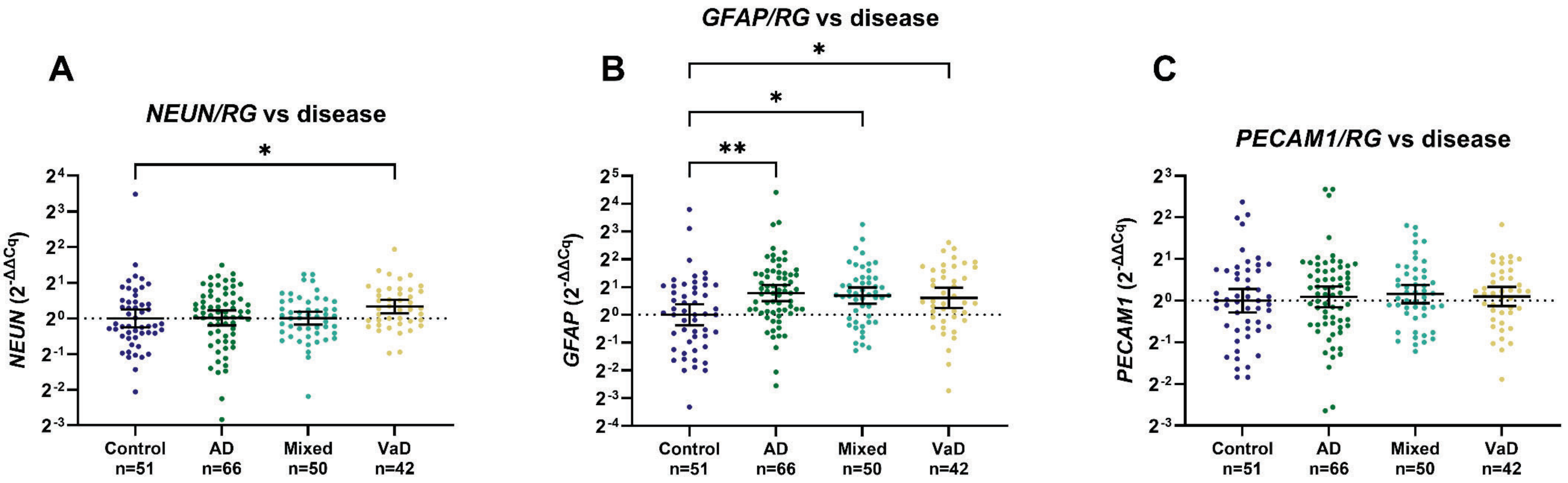

Supplementary Figure 12. Gene expression of cell-specific markers in the frontal cortex in disease. Gene expression was measured by RT-PCR in a cohort (n = 209) divided into diagnoses groups: age-matched controls (n = 51), Alzheimer's disease (AD) (n = 66), AD and VaD (mixed) (n = 50) and Vascular dementia (VaD) (n = 42). Cell-specific gene markers (A. *NEUN*, B. *GFAP*, C. *PECAM1*) were calibrated to reference genes and expressed using the $2^{-\Delta\Delta Cq}$ method. Individual dots represent an individual case measured in triplicate. The geometric mean and 95% confidence interval are shown. *p<0.5, **p<0.001.

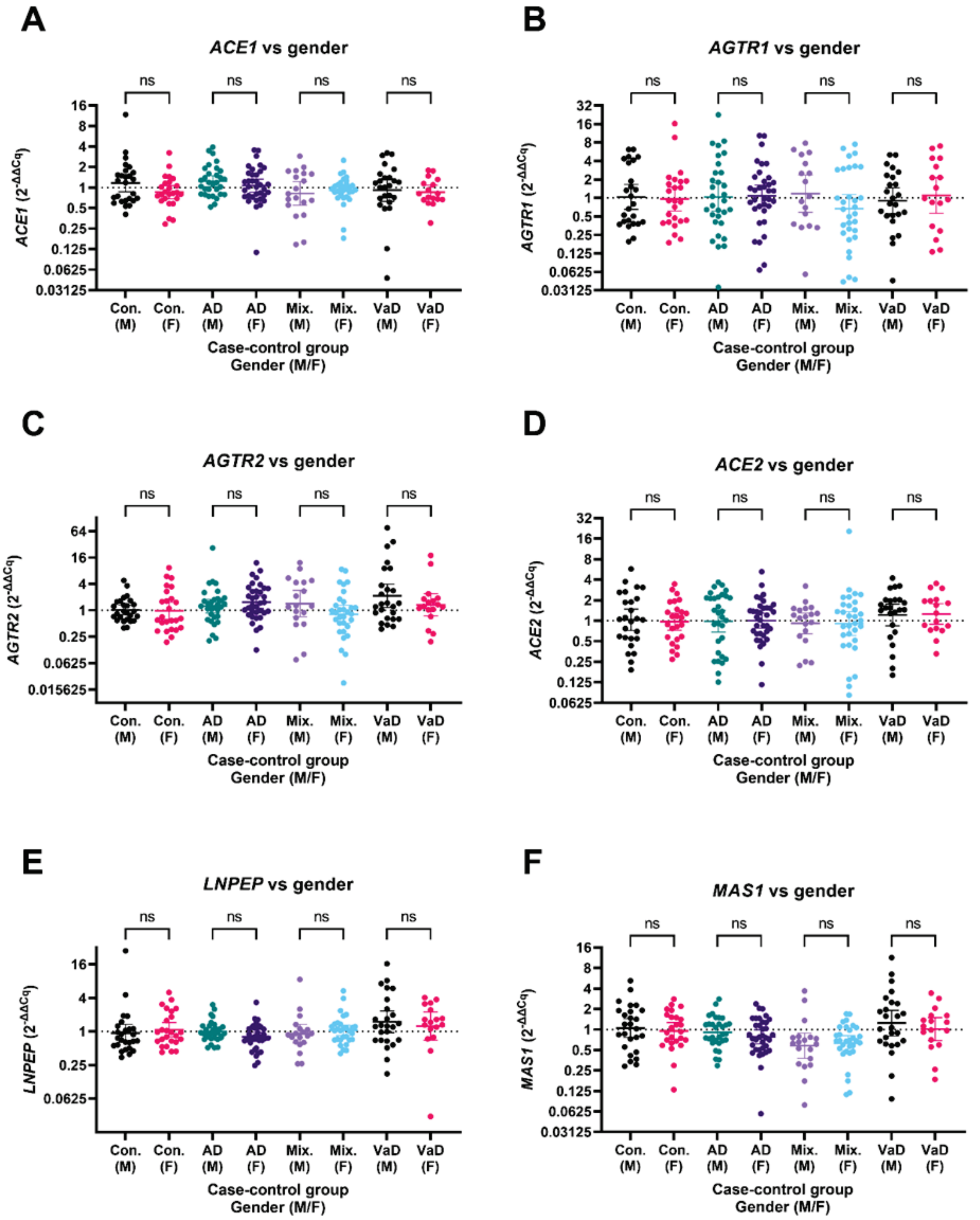

Supplementary Figure 13. Absence of gender-specific alterations in RAS gene expression in dementia. No statistically significant effects of gender were observed in dementia groups: AD, AD/VaD mixed, VaD, or the age-matched controls. Data were analysed using Kruskal-Wallis with Dunn's posthoc test. Each data point represents gene expression from one individual donor.

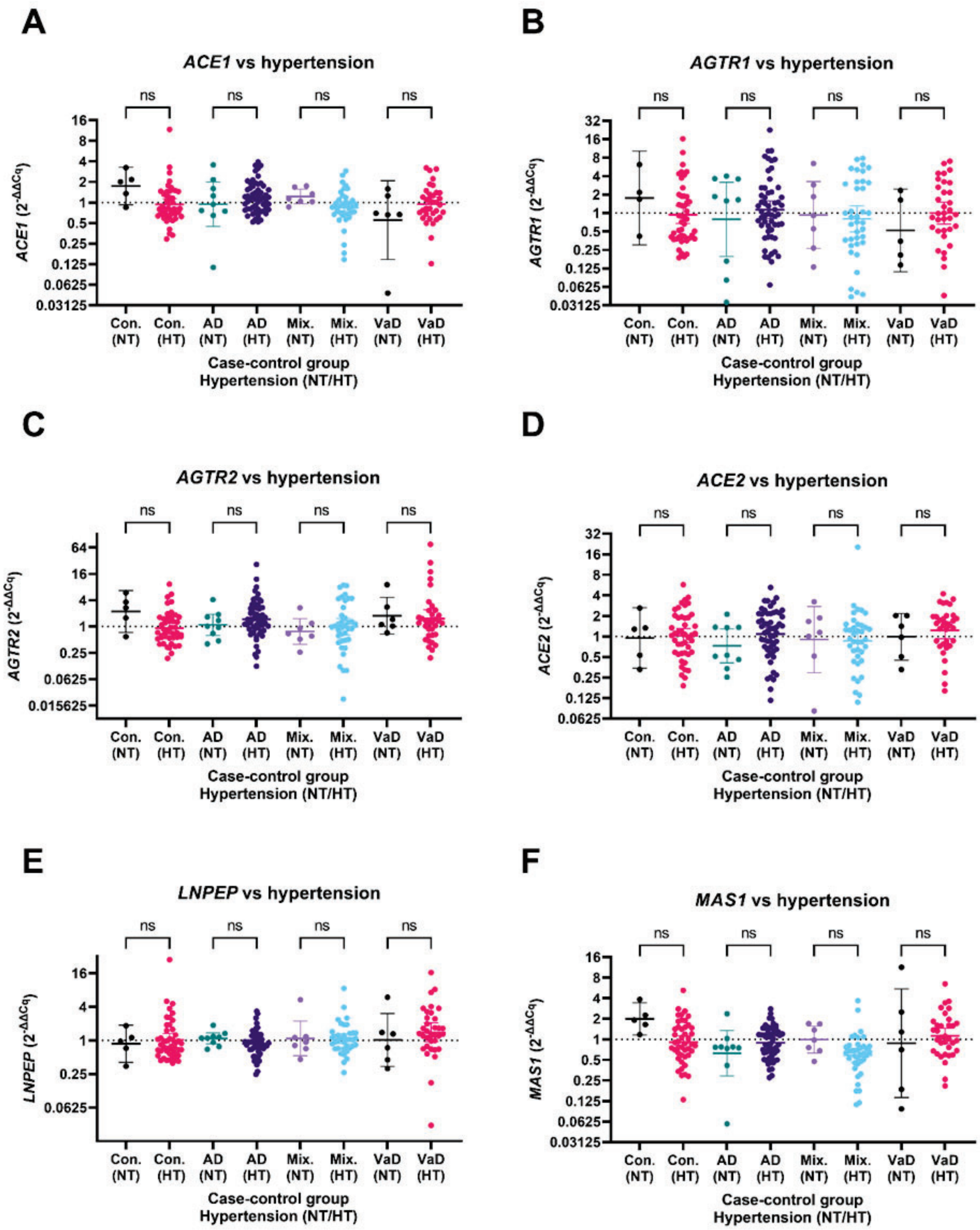

Supplementary Figure 14. Hypertension status does not influence RAS gene expression in dementia. No statistically significant differences in RAS gene expression were observed in the hypertensive (HT) Vs normotensive (NTs) groups in each of the dementia groups: AD, AD/VaD mixed, VaD, or the age-matched controls. Data were analysed using Kruskal-Wallis with Dunn's posthoc test. Each data point represents gene expression from one individual donor.

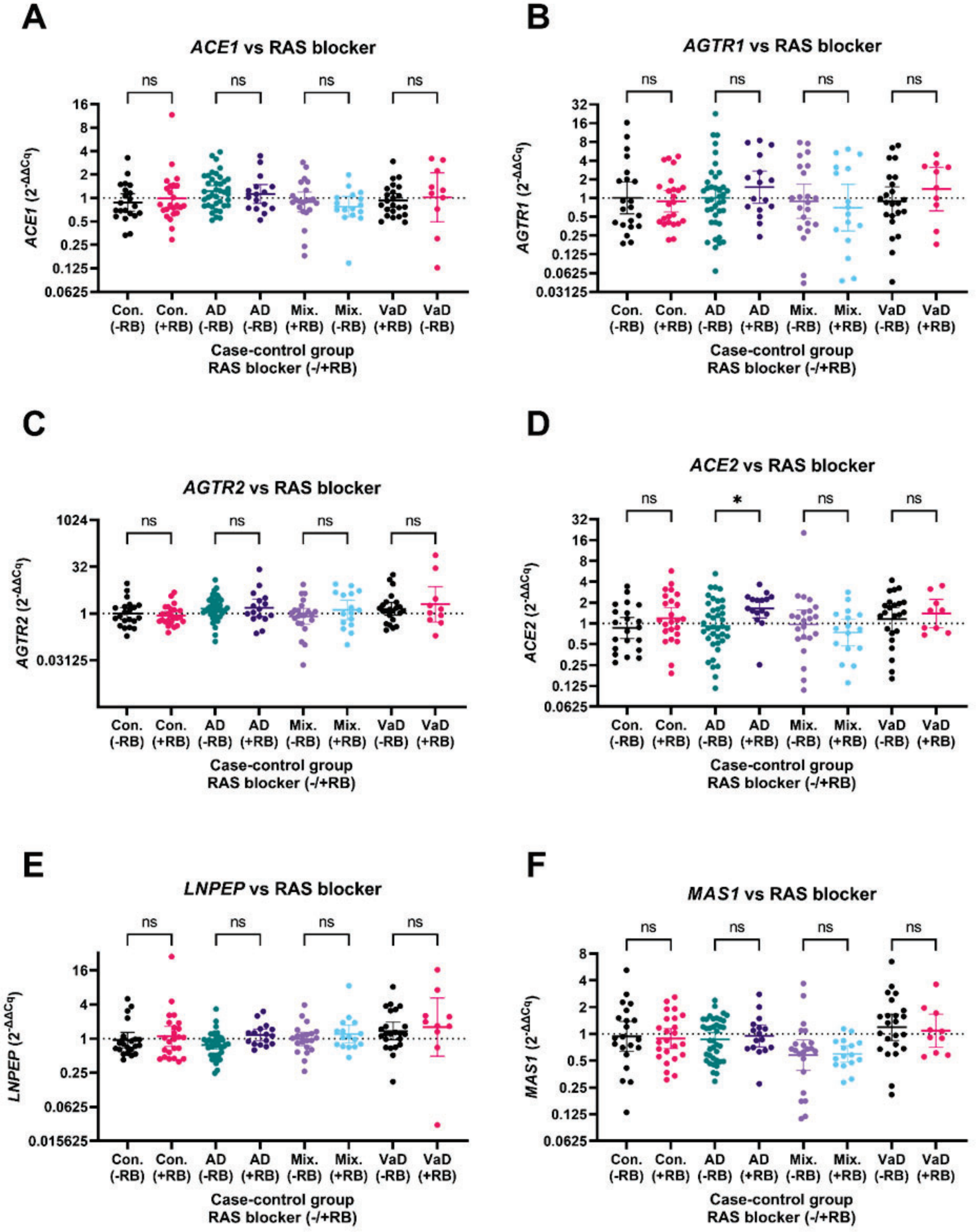

Supplementary Figure 15. A history of RAS medication is associated with elevated ACE2 gene expression in Alzheimer's disease. No statistically significant effects of RAS-targeting medication (+RB) on RAS gene expression were observed in each of the dementia groups: AD, AD/VaD mixed, VaD, or the age-matched controls except for higher *ACE2* expression is higher in the RAS-targeting subgroup in the AD group (Kruskal-Wallis with Dunn's posthoc test; p=0.0495). Data were analysed using Kruskal-Wallis with Dunn's posthoc test. Each data point represents gene expression from one individual donor.
